# Nanocomposite Hydrogel for Real‐Time Wound Status Monitoring and Comprehensive Treatment

**DOI:** 10.1002/advs.202405924

**Published:** 2024-09-13

**Authors:** Huan Lei, Xueqing Yu, Daidi Fan

**Affiliations:** ^1^ Engineering Research Center of Western Resource Innovation Medicine Green Manufacturing Ministry of Education School of Chemical Engineering Northwest University Xi'an 710069 China; ^2^ Shaanxi Key Laboratory of Degradable Biomedical Materials and Shaanxi R&D Center of Biomaterials and Fermentation Engineering School of Chemical Engineering Northwest University Xi'an 710069 China; ^3^ Biotech. & Biomed. Research Institute Northwest University Xi'an 710069 China

**Keywords:** diabetic wounds, nanocomposite hydrogel, real‐time monitoring, wound repair

## Abstract

Current skin sensors or wound dressings fall short in addressing the complexities and challenges encountered in real‐world scenarios, lacking adequate capability to facilitate wound repair. The advancement of methodologies enabling early diagnosis, real‐time monitoring, and active regulation of drug delivery for timely comprehensive treatment holds paramount significance for complex chronic wounds. In this study, a nanocomposite hydrogel is devised for real‐time monitoring of wound condition and comprehensive treatment. Tannins and siRNA containing matrix metalloproteinase‐9 gene siRNA interference are self‐assembled to construct a degradable nanogel and modified with bovine serum albumin. The nanogel and pH indicator are encapsulated within a dual‐crosslinking hydrogel synthesized with norbornene dianhydride‐modified paramylon. The hydrogel exhibited excellent shape adaptability due to borate bonding, and the click polymerization reaction led to rapid in situ curing of the hydrogel. The system not only monitors pH, temperature, wound exudate alterations, and peristalsis during wound healing but also exhibits hemostatic, antimicrobial, anti‐inflammatory, and antioxidant properties, modulates macrophage polarization, and facilitates vascular tissue regeneration. This therapeutic approach, which integrates the monitoring of pathological parameters with comprehensive treatment, is anticipated to address the clinical issues and challenges associated with chronic diabetic wounds and infected wounds, offering broad prospects for application.

## Graphical abstract

A nanocomposite dual network hydrogel system is designed for real‐time monitoring of wound status and comprehensive therapy, enabling the surveillance of pH, temperature, alterations in wound exudate, and mobility, and dynamically regulating the microenvironment throughout the wound healing process, thereby fostering the recovery of infected wounds. This is anticipated to address the clinical issues and challenges associated with chronic diabetic wounds.

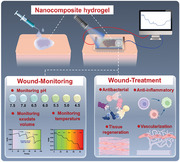

## 1 Introduction

Chronic wounds and the consequent wound infections pose substantial medical challenges, encompassing tissue damage, organ dysfunction, and sepsis, thereby exerting a profound impact on the lives of patients.^[^ ^1^ ^]^ Hence, timely surveillance and management of infected wounds are imperative for clinical care. In recent times, flexible electronics have garnered considerable attention in the advancement of diverse wearable healthcare systems for detecting physiological and biochemical parameters,^[^ ^2^ ^]^ providing distinctive solutions for the surveillance and management of ailments such as chronic and infected wounds.

In recent years, propelled by the advancement of smart materials adept at responding to diverse external stimuli, a plethora of smart wound dressings have been extensively researched and developed for wearable wound management.^[^ ^3^ ^]^ For instance, Qingjun Liu et al.^[^ ^4^ ^]^ devised intelligent dressings capable of concurrently detecting wound temperature, pH, and uric acid levels, with drug‐delivery electrodes embedded in the dressings for on‐demand infection treatment via an electronically controlled antibiotic release. Lei Zhang et al.^[^ ^5^ ^]^ engineered skin sensors enabling continuous real‐time monitoring of multiple parameters (temperature, strain, and glucose concentration). Lie Ma et al.^[^ ^6^ ^]^ formulated skin dressings for real‐time wound temperature monitoring, facilitating early infection diagnosis and on‐demand infection treatment by antibiotics released from hydrogels through in situ UV irradiation. Nevertheless, many of these advancements are limited to sensor integration, failing to adequately address the challenges encountered in real‐world applications, and exhibiting insufficient capability for wound repair.

When the integrity of the skin is compromised, wounds become vulnerable to infection by pathogenic bacteria, particularly in the case of diabetic wounds.^[^ ^7^ ^]^ Bacterial infestation induces tissue necrosis, triggering the continuous production of matrix metalloproteinases and toxins, culminating in prolonged inflammation and hindered wound healing.^[^ ^8^ ^]^ The second stage of wound healing entails inflammation, marked by heightened expression of pro‐inflammatory macrophages with elevated levels of ROS, exacerbating local inflammation and impeding angiogenesis.^[^ ^9^ ^]^ The third phase of wound healing, known as the proliferative phase, involves cell migration to the wound site and the process of neovascularization.^[^ ^10^ ^]^ Nutrients are transported to the impaired area via the blood vessels, facilitating the reparative process.^[^ ^11^ ^]^ The intricacy of the microenvironment during the wound healing process imposes rigorous demands on treatment modalities. However, existing approaches either concentrate on individual stages with restricted therapeutic efficacy or lack the ability to autonomously adjust to the intricacies of the wound microenvironment. Hence, the development of methodologies enabling early diagnosis, real‐time monitoring, and active drug delivery control for timely and comprehensive treatments is of paramount importance for managing complex chronic wounds.

**Scheme 1:**
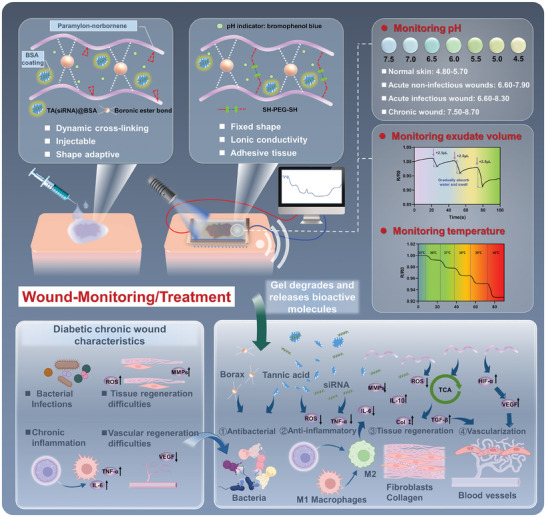
Conceptualization of a nanocomposite hydrogel for real‐time monitoring of wound status and holistic healing.

In accordance with the aforementioned requirements, this study developed a nanocomposite hydrogel for real‐time monitoring of wound status and comprehensive therapy, enabling the surveillance of pH, temperature, alterations in wound exudate, and mobility, and dynamically regulating the microenvironment throughout the wound healing process, thereby fostering the recovery of infected wounds (**Scheme** **1**). A degradable nanogel was synthesized through the self‐assembly of tannins and siRNA loaded with MMP‐9 gene‐interfering siRNA. Bovine serum albumin (BSA) coating was employed to enhance the stability and biocompatibility of the nanogel. The nanogel, along with the pH indicator bromothymol blue, was encapsulated within a dual‐crosslinking hydrogel polymerized with norbornene dianhydride‐modified paramylon (N‐P). The initial layer of the flexible crosslinking network, facilitated by borate bonding, maintains the hydrogel in a state between sol and gel, enabling the nanogel‐loaded hydrogel precursor to be injected onto the wound surface for full adhesion. Subsequently, a click polymerization reaction is utilized to enable N‐P to cure in situ in the presence of a cross‐linking agent under light conditions, rapidly forming a second crosslinking that serves as a hemostatic barrier and facilitates adhesion of the hydrogel to the wound surface. The system can accomplish the following functionalities: 1) Wound monitoring–the color changes of bromothymol blue signify variations in wound pH, while borax confers ionic conductivity to the hydrogel for monitoring exudate volume and wound temperature changes. 2) Promotion of chronic wound repair–The second layer of crosslinks, cured in situ, serves as a sealing and hemostatic agent. The presence of borax and TA in the nanogel endowed the hydrogel with potent antimicrobial properties. The siRNA released from the nanogel can attenuate the expression of MMP‐9 in the wound. TA and paramylon can synergize to impart anti‐inflammatory and antioxidant effects, promoting macrophage polarization and tissue regeneration. Moreover, paramylon promotes VEGF expression and vascularization. In conclusion, the platform proposed in this study lays the groundwork for the development of more advanced wearable medical systems for monitoring additional pathological parameters and facilitating comprehensive treatments, thereby holding promise for applications in the biomedical field.

## 2 Results and Discussion

### 2.1 Design, Synthesis, and Characterization of the TA‐siRNA@BSA Nanogel

**Figure 1:**
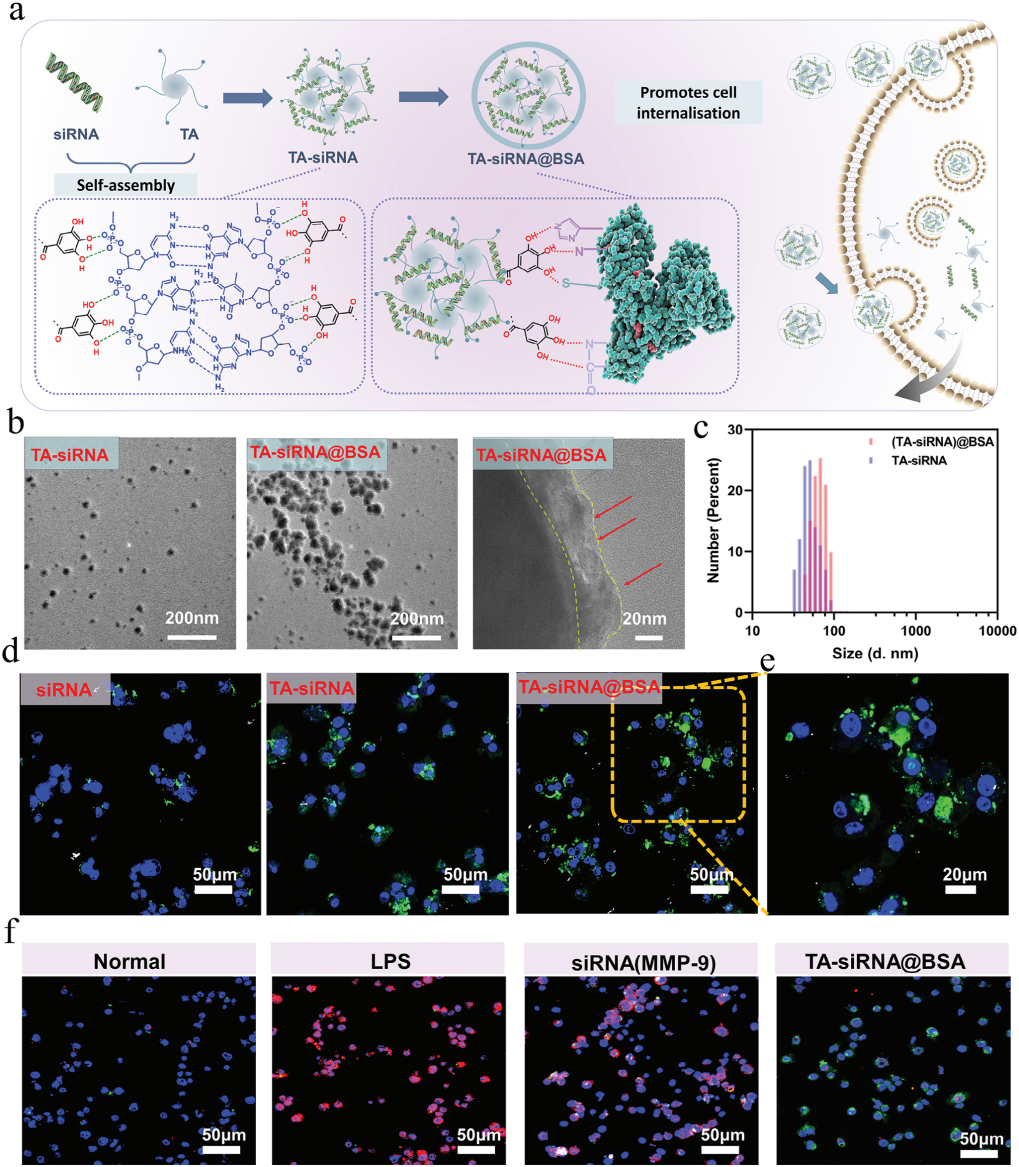
Preparation and gene silencing efficiency of TA‐siRNA @BSA nanogel, a) Schematic diagram of the formation mechanism of TA‐siRNA @BSA nanogel; b) TEM of TA‐siRNA and TA‐siRNA @BSA nanogel; c) Nanogel size distribution; d) Confocal microscopy images of macrophages after the cellular uptake of siRNA, TA‐siRNA and TA‐siRNA@BSA nanogels. The scale bar represents a 50 µm scale. e) the high‐magnification confocal microscopy images. The siRNA was labeled with a green 5‐carboxyfluorescein (5‐FAM). The nucleus of the cells was labeled with DAPI (blue). The scale bar represents a 20 µm scale; f) Gene silencing efficiency of TA‐siRNA nanogels in RAW264.7 cell. siRNA was labeled with 5‐FAM, and MMP‐9 expression was assessed using immunofluorescence staining. Cell nuclei were labeled with DAPI (blue). The scale bar is 50 µm.

To mitigate the overexpression of MMP‐9 in diabetic wounds, we devised and fabricated a nanogel loaded with small interfering RNA (**Figure** **1**a). The hydrogen bonding between siRNA and TA facilitated the self‐assembly process, resulting in the formation of TA‐siRNA nanogel. Moreover, the catechol structure of TA could engage in reversible hydrogen bonding with the phosphate backbone of siRNA, leading to the release of siRNA along with the hydrolysis of ester bonds. In a prior investigation, we elucidated the cross‐linking mechanism and siRNA release kinetics of TA‐siRNA nanogel,^[^ ^9^ ^]^ Nevertheless, this nanogel is easily hydrolyzed in aqueous environments, and the TA released will cause the environment to become acidic, which is not beneficial to the growth of cells. In this investigation, BSA was employed to create coatings on TA‐siRNA nanogels. These nanogels possess numerous phenolic groups on their surface, capable of cross‐linking with specific protein groups. The application of BSA coating improves the stability in aqueous environments and biocompatibility of the nanogel.^[^ ^12^ ^]^ We observed the morphology of the nanogel by TEM after 6 h in an acidic environment, the TA(siRNA)@BSA nanogel showed degradation, but part of the gel could still be observed, whereas the TA(siRNA) was almost completely degraded, suggesting that the BSA enhanced its stability (Figure S2, Supporting Information). TA is an acidic substance, which causes a change in the pH of the cell culture solution at higher concentrations, and thus a lower cell survival rate occurs at high concentrations. In addition, the BSA coating improves the stability of the nanogel and has a slow‐release effect, and the release of TA is delayed, so the cell survival rate is increased. And since BSA is a nutrient, it helps in cell proliferation. Therefore, the slow‐release effect provided by the BSA coating as well as the cell‐proliferative effect of BSA can alleviate the damage of TA on cells (Figure S3, Supporting Information). The morphology of both TA‐siRNA nanogel and TA‐siRNA@BSA can be visualized using transmission electron microscopy (TEM) (Figure 1b), and the coating results in an enlargement of the gel size, with the size of TA‐siRNA@BSA measuring ≈100 nm (Figure 1c). The cellular uptake rates (Figure S4, Supporting Information) of the nanogels were measured to be 7.35 ± 6.36% (siRNA), 56.73 ± 5.34% (TA‐siRNA), and 84.27 ± 3.52% (TA‐siRNA@BSA). Notably, the TA‐siRNA@BSA nanogel carrier exhibits enhanced cell internalization compared to both siRNA and TA‐siRNA nanogels (Figures 1d,e). The gene silencing efficiency of the TA‐siRNA@BSA nanogel is depicted in Figure 1f and Figures S1,S18 (Supporting Information). LPS was employed to stimulate a substantial expression of MMP‐9 in macrophages. Notably, a relatively intense green fluorescence was observed surrounding the cells in the TA‐siRNA@BSA (MMP‐9) nanogel group, indicating an enhanced cellular internalization effect. Moreover, the MMP‐9 level in the cells treated with the TA‐siRNA@BSA (MMP‐9) nanogel was significantly lower compared to that in the LPS and siRNA (MMP‐9) group, underscoring the robust gene silencing efficiency of the TA‐siRNA@BSA (MMP‐9) nanogel. Subsequently, we incorporated this nanogel into a hydrogel with a TA‐siRNA@BSA nanogel content of 0.2 OD 1 mL^−1^ of hydrogel. The determination of the TA‐siRNA@BSA nanogel content in the hydrogel was based on the effective siRNA concentration established through cell internalization and gene silencing experiments.

### 2.2 Design, Synthesis, and Characterization of Multifunctional Hydrogel

**Figure 2:**
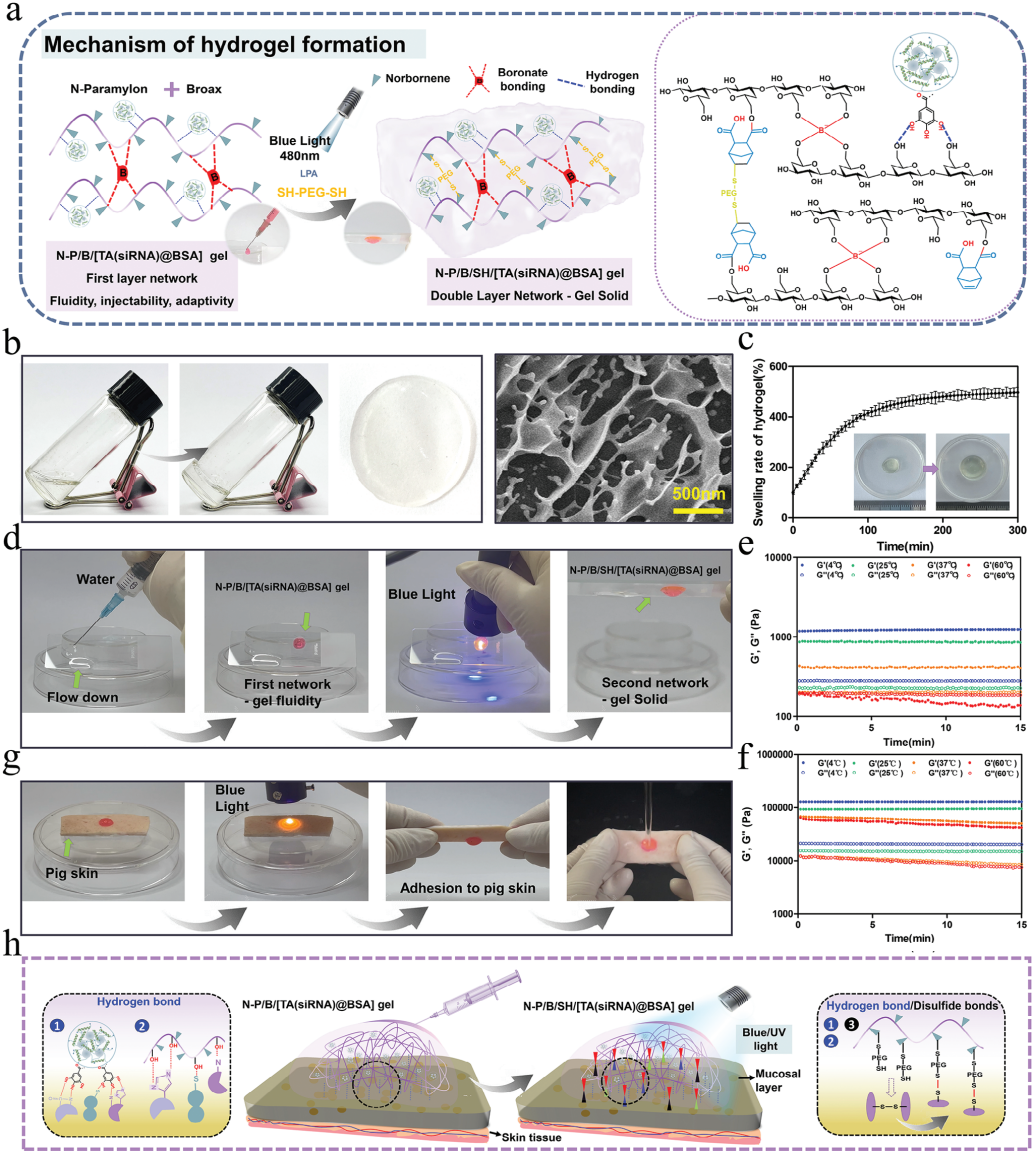
Physical and chemical properties of hydrogels: a) Schematic diagram of hydrogel cross‐linking mechanism; b) Macroappearance and microstructure of the N‐P/B/SH/[TA‐siRNA)@BSA] hydrogel after blue‐light irradiation; c)Swelling of N‐P/B/SH/[TA(siRNA)@BSA] hydrogel; d)Hydrogel formation process and gel state (Rhodamine dye added to hydrogels to enhance visibility); e) Rheodynamics : amplitude‐time scan (strain 1%) of N‐P/B/ [TA(siRNA)@BSA] hydrogel; f) Rheodynamics : amplitude‐time scan (strain 1%) of N‐P/B/SH/[TA(siRNA)@BSA] hydrogel; g) Hydrogel formation process and adhesion; h) Schematic representation of the adhesion mechanism (Rhodamine dye added to hydrogels to enhance visibility).

The cross‐linking mechanism of the hydrogel is illustrated in **Figure** **2**a. Initially, norbornene was grafted onto paramylon (as depicted in Figures S5–S7, Supporting Information), followed by blending with TA‐siRNA@BSA nanogel, lithium phenyl‐2,4,6‐trimethylbenzoylphosphite (LAP), and bipartite mercapto polyethylene glycol (SH‐PEG‐SH), with the subsequent addition of borax. The primary layer of the crosslinking network comprises a borate bond formed between borax and paramylon, characterized by its dynamic nature, enabling the hydrogel to oscillate between sol and gel states, thereby facilitating adequate wound accommodation and gap filling. Subsequently, blue light irradiation was employed to trigger the click polymerization reaction, enabling N‐P to swiftly form a secondary crosslinking network layer via in situ curing in the presence of a cross‐linking agent under light conditions, thereby promoting adhesion of the hydrogel to the wound surface.

The hydrogel exhibited a transparent appearance (as illustrated in Figure 2b; Figure S8, Supporting Information), while microstructural analysis revealed its porous nature, laden with numerous nanogel‐like spheres on the paramylon matrix. The swelling behavior of the hydrogel is delineated in Figure 2c, showcasing a fourfold increase in swelling rate and a 4.45‐fold increase in volume at equilibrium. Such remarkable swelling performance establishes a solid foundation for wound exudate monitoring.

The distinction between the two hydrogel networks is depicted in Figure 2d, where water cascades down the glass bevel. However, the gel containing the initial layer crosslinking network (borate bonding) exhibits the ability to adhere to the glass bevel post‐injection, descending at a markedly sluggish pace. Within 30–60s of blue light exposure, the secondary crosslinking network of the hydrogel (via click polymerization reaction of thiols‐alkenes) is initiated, resulting in complete solidification of the hydrogel.

Rheomechanical analysis was employed to investigate the hydrogel states under varying temperatures (Figures 2e,f; Figure S9, Supporting Information). The N‐P/B/[TA(siRNA)@BSA] hydrogel comprises solely the initial layer of the crosslinking network. At 4, 25, and 37 °C, G′ surpasses G″, indicative of the hydrogel's solid gel state. However, with rising temperature, G′ gradually diminishes, attributed to the partial disruption of borate bonds due to thermal escalation. Notably, at 60 °C, G′ falls below G″, suggesting the hydrogel's transition to fluidic properties, indicative of extensive borate bond rupture at this temperature.

In contrast to the N‐P/B/[TA(siRNA)@BSA] hydrogel, the N‐P/B/SH/[TA(siRNA)@BSA] hydrogel exhibited sustained solidity (depicted in Figure 2f). Notably, even at an elevated temperature of 60 °C, G′ surpassed G″, attributable to the persistence of the second layer crosslinking network within the hydrogel. Analogous to the N‐P/B/[TA(siRNA)@BSA] hydrogel, a gradual reduction in G′ with temperature elevation ensued, primarily attributed to borate bond cleavage. Furthermore, akin to the mechanical properties, the thermogravimetric curves also delineate the impact of the two distinct crosslinking networks on the thermal stability of the hydrogel (as depicted in Figure S10, Supporting Information).

The adherence of the hydrogel to pig skin is illustrated in Figure 2g. Following blue light irradiation, the N‐P/B/SH/[TA(siRNA)@BSA] hydrogel exhibits adhesion to pig skin, with an adhesion strength reaching 37 ± 3.2 kPa (as indicated in Figure S11, Supporting Information). Notably, it remains firmly adhered even under the influence of water flow, attributed to the hydrogel's capacity to form hydrogen or disulfide bonds with the tissue (as depicted in Figure 2h), thereby facilitating tissue adhesion.

The siRNA release rate (Figure S12, Supporting Information) of TA(siRNA)@BSA hydrogel was significantly lower than that of TA(siRNA), indicating that the BSA coating could enhance the structural stability of the hydrogel. In addition, the release rate of siRNA in an acidic environment (pH 4.0) was significantly higher than that in a neutral environment (pH 7.0), which suggests that it can release functional substances under acidic (wound site) conditions to exert the antioxidant, antibacterial, and scavenging efficacy of MMP‐9. TA is mainly dissociated from the nanogel, and TA is hydrolyzed to Gallic acid (GA) and Ellagic acid (EA) when the ester bond is broken^[^ ^13^ ^]^ in an aqueous environment (Figure S13, Supporting Information), and this degradation and dissociation is faster in an acidic environment. Interestingly, the release of GA/EA is larger than that of TA within 2 h, indicating that in the early stage, a part of the ester bond of TA in the nanogel breaks and releases a part of GA/EA, while in the later stage, the overall hydrogen bond of the gel dissociates and releases TA and the ester bond of the released TA breaks further and releases GA and EA. The nanogel loaded with hydrogel can have a more stable and sustained release of TA than nanogel.

In addition, the adsorption performance of the hydrogel for bovine serum albumin was also measured (Figure S14, Supporting Information), and the results showed that the adsorption of N‐P/B/SH hydrogel reached equilibrium at ≈80 min. The adsorption effect and adsorption rate of N‐P/B/SH/[TA(siRNA)@BSA] hydrogel were lower than that of N‐P/B/SH hydrogel, possibly because TA(siRNA)@BSA nanogel occupied part of the adsorption site.

### 2.3 Antibacterial and Antioxidant Properties

**Figure 3:**
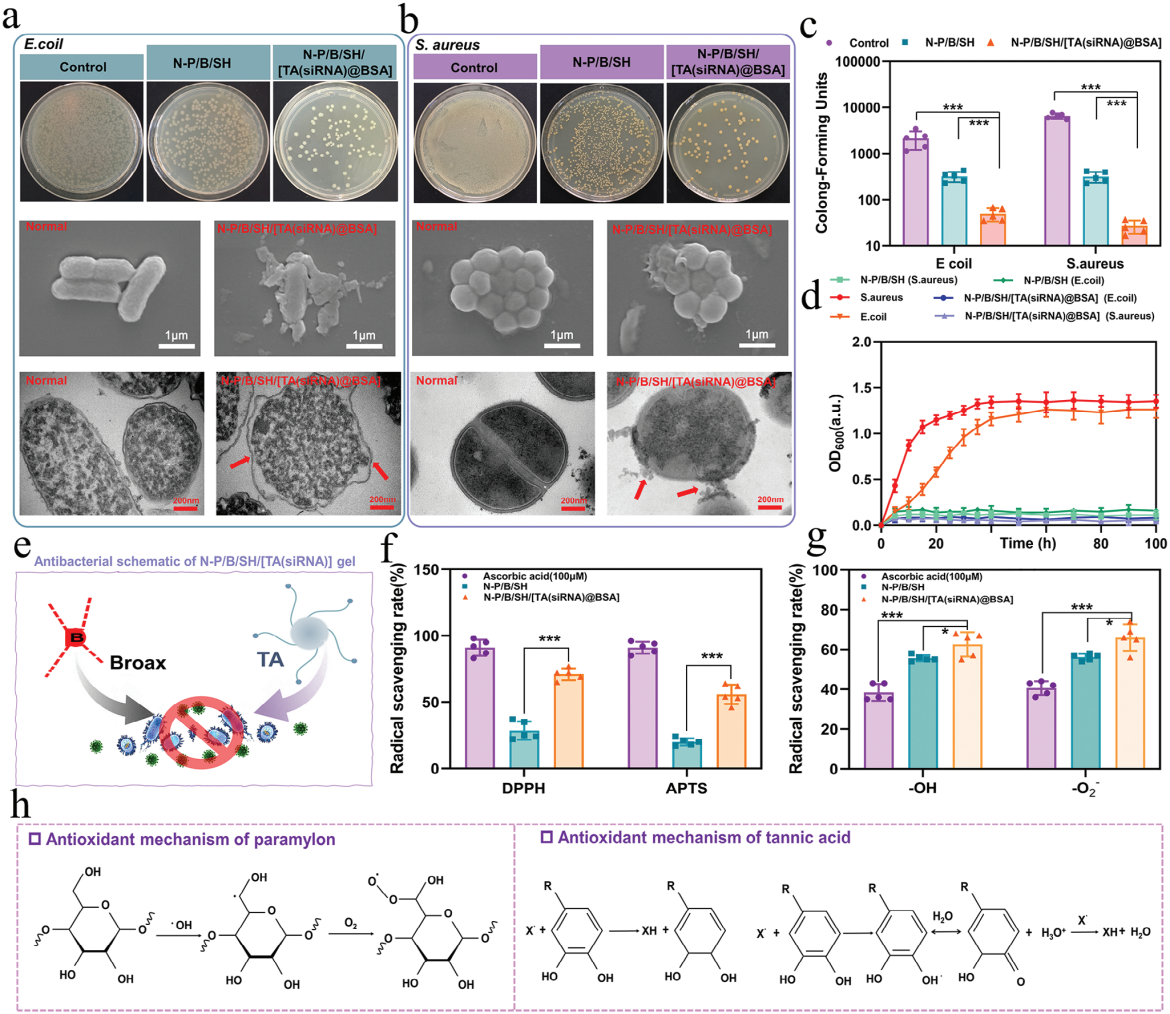
Antibacterial and antioxidant properties: a) Antibacterial–*Escherichia coli* (plate counting method; scanning electron microscopy (SEM), TEM); b) Antibacterial–*Staphylococcus aureus* (plate counting method; SEM, TEM); c) Quantitative data on colony‐forming units by plate counting method; d) Bacterial density of *E. coli* and *S. aureus* in LB liquid medium; e) Antibacterial Schematic; f) The scavenging effects of hydrogels on the DPPH radical and ABTS^∙+^ radical; g) The scavenging effects of hydrogels on the ‐OH and ‐O_2_ radical; h) Schematic representation of the antioxidant mechanism. ^*^ *p* < 0.05, ^***^ *p* < 0.001, *n* = 5.

Diabetic wounds, predisposed to infection by pathogenic bacteria,^[^ ^14^ ^]^ necessitate the integration of components such as borax and tannic acid into the hydrogel system to confer antiseptic properties, thereby combating pathogenic bacteria. **Figures** **3**a,b depicts the experimental outcomes of the standard plate method following the co‐culture of *E. coli* and *S. aureus* with the hydrogel. Remarkably, the control group displays a noteworthy bacterial count. In contrast to the control, the N‐P/B/SH/[TA(siRNA)@BSA] hydrogel group exhibited a substantial reduction in colony numbers, with quantitative analysis (as depicted in Figure 3c) demonstrating an inhibition efficiency exceeding 96% against *E. coli* and over 99% against *S. aureus*, showcasing its remarkable antibacterial efficacy. Similarly, the colony count decreased considerably in the N‐P/B/SH hydrogel group, with inhibition efficiencies surpassing 82% against *E. coli* and over 94% against *S. aureus* (as illustrated in Figure 3c). The OD600 values of the *E. coli* and *S. aureus* blank groups without hydrogel increased over the incubation period (depicted in Figure 3d) and stabilized thereafter, consistent with the growth pattern of normal bacteria. Both the N‐P/B/SH/[TA(siRNA)@BSA] hydrogel and N‐P/B/SH hydrogel markedly inhibited the growth of *E. coli* and *S. aureus*, with their antimicrobial activities predominantly attributed to TA and borax (illustrated in Figure 3e). The results showed that there was no significant difference between N‐P/B/SH/[TA(siRNA)@BSA] and N‐P/B/SH/[TA(siRNA)@BSA]+bromophenol blue hydrogel groups(Figure S15, Supporting Information). The concentration of bromophenol blue in the hydrogel was 0.1 mg mL^−1^, which had no significant effect on the antibacterial properties at this lower concentration and with the slow release of the hydrogel. Scanning electron microscopy (SEM) and TEM observations revealed morphological alterations in the two bacteria (as depicted in Figures 3a,b).

In the blank group, *S. aureus* displayed a uniform spherical morphology, while *E. coli* exhibited characteristic rod‐like structures with intact and smooth cell membrane structures. Conversely, within the N‐P/B/SH/[TA(siRNA)@BSA] hydrogel group, evident disruption of the bacterial cell membrane structure was observed in numerous instances, accompanied by significant intracellular material leakage, indicating a discernible bactericidal effect of the N‐P/B/SH/[TA(siRNA)@BSA] hydrogel.

A noteworthy characteristic of diabetic wounds is the aberrant glycolipid metabolism, resulting in an excessive generation of ROS, which not only instigates a potent inflammatory response but also impairs macrophage function, thereby hindering the regeneration of wound tissue.^[^ ^15^ ^]^ Furthermore, ROS notably impede angiogenesis and induce endothelial dysfunction, which can directly or indirectly compromise the functionality of cells such as keratinocytes and dermal fibroblasts, thereby culminating in challenges associated with wound healing.^[^ ^16^ ^]^

Hence, the antioxidant characteristics of the dressing hold significant importance. We investigated the in vitro antioxidant properties of the N‐P/B/SH/[TA(siRNA)@BSA] hydrogel through assays targeting 2,2‐Diphenyl‐1‐picrylhydrazyl (DPPH), 2,2‐Biazobis(3‐ethylbenzothiazole‐6‐sulfonic acid) diammonium salt radicals (ABTS^·+^), ‐OH, and ‐O_2_ scavenging. Illustrated in Figures 3g,h, we employed ascorbic acid as a positive control to evaluate the antioxidant attributes of the hydrogel. Ascorbic acid, widely acknowledged as the most extensively used antioxidant, features an enediol moiety in its molecular structure, facilitating its conversion to ketone groups to neutralize free radicals. Remarkably, the free radical scavenging efficacy of the N‐P/B/SH/[TA(siRNA)@BSA] hydrogel augmented with TA(siRNA)@BSA nanogel markedly surpassed that of the N‐P/B/SH hydrogel, underscoring the significant role of TA's antioxidant prowess in this context. Furthermore, it was observed that both hydrogels exhibited superior scavenging capability toward ‐OH and ‐O_2_ radicals compared to DPPH, ABTS^·+^ radicals, and even surpassing that of 100 µm ascorbic acid. Our previous investigations have illustrated that paramylon can exert a direct impact on the annihilation of free radicals, deoxygenation, and attenuation of oxygen in the singular‐linear state. Hence, the scavenging efficacy against oxygen radicals may be ascribed to both paramylon and TA, whereas for the scavenging of DPPH, ABTS^·+^ radicals, it is conjectured that solely TA in the nanogels may play a contributory role. The polysaccharides can regulate the expression of downstream antioxidant enzymes through the endogenous anti‐oxidative stress Nrf2/ARE pathway, in addition to immediately scavenging the generated ROS.^[^ ^17^ ^]^ Tannins are esters formed by combining the 5 hydroxyl groups of glucose and galloyl groups and belong to the hydrolyzed tannins, which can be hydrolyzed to glucose and gallic acid. Based on the structure of tannins, it is difficult for them to enter the cell through the cell membrane, and the molecular mechanism by which tannins achieve transmembrane signaling is still unclear. It has been shown that TA increases the activities of SOD, POD, CAT, and APX enzymes, which are important components of the antioxidant defense system, and increases the accumulation of proline and GSH, both of which are involved in scavenging free radicals and reducing oxidative stress.^[^ ^18^ ^]^ How TA removes ROS through direct or indirect actions remains a controversial issue, and further research is necessary to fully understand the mechanisms involved.

### 2.4 Cytocompatibility, Cell Migration, Tubule Formation Assay, Macrophage Polarization

**Figure 4:**
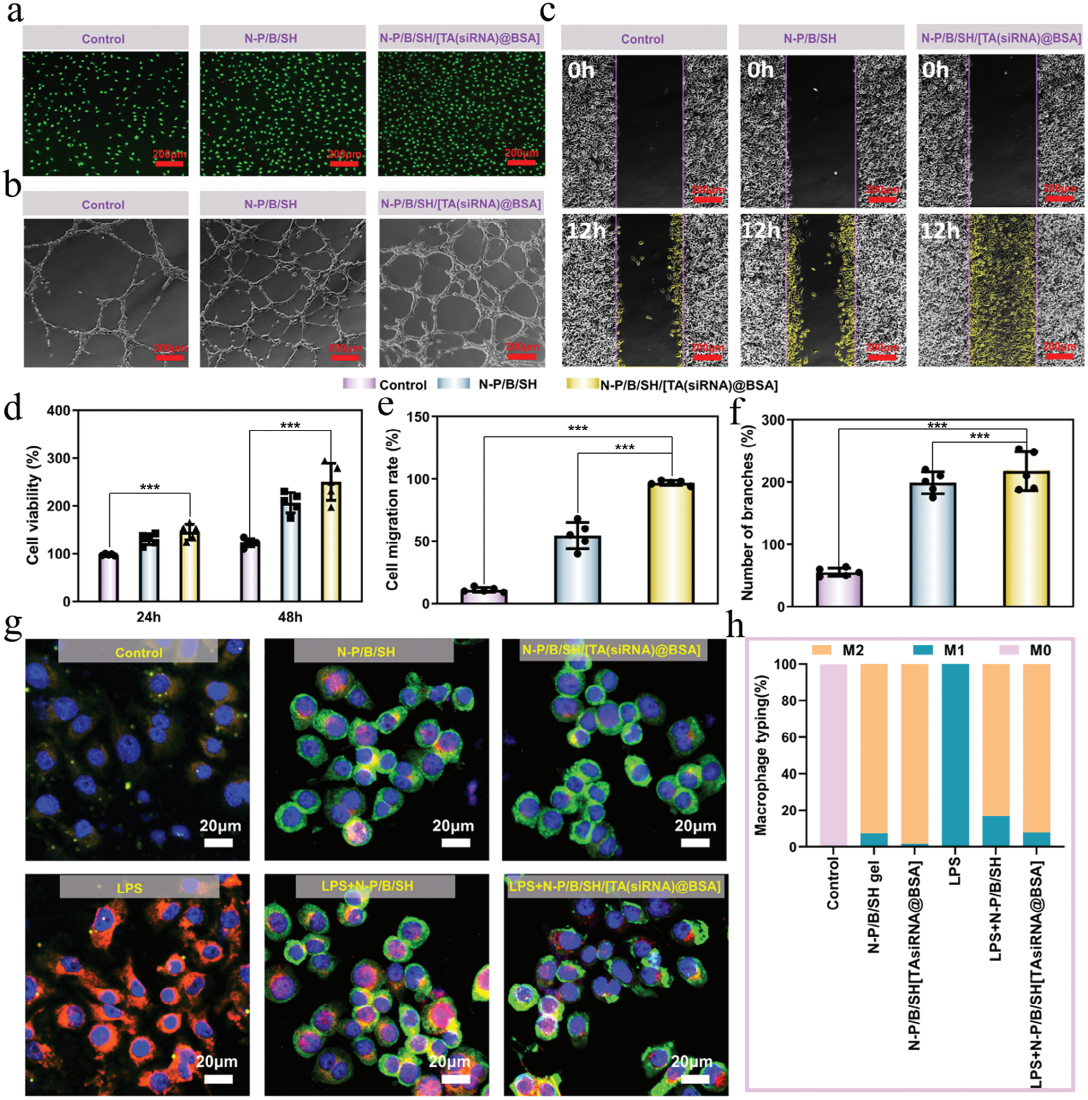
Cytological experiments: a) Live‐dead cell staining (AO/EB) of L929 cells cultured with hydrogel extracts (scale bar: 200 µm); b) Images of tube formation by HUVECs(scale bar: 200 µm); c) L929 cell migration(scale bar: 200 µm); d) Cell viability with the hydrogel extracts; e) quantitative data on cell mobility; f) quantitative data on the number of branch points in angiogenesis experiments; g) The immunofluorescent staining of CD86‐labeled M1 macrophages (red staining) and CD206‐labeled M2 macrophages (green staining); h) Population distribution of M0, M1 and M2 macrophages. ^***^ *p* < 0.001, *n* = 5.

The cytocompatibility of the hydrogels was thoroughly assessed through AO/EB live‐dead staining and MTT experiments (**Figures** **4**a,d). AO/EB staining was meticulously examined under a fluorescence microscope, revealing a predominant presence of green fluorescence among almost all cells. Notably, the cell density in the N‐P/B/SH/[TA(siRNA)@BSA] hydrogel group surpassed that of both the control and N‐P/B/SH hydrogel groups. Furthermore, MTT assay results illustrated that the cell survival rate for both N‐P/B/SH/[TA(siRNA)@BSA] hydrogel and N‐P/B/SH hydrogel exceeded 100% and exhibited an upward trajectory with prolonged culture duration, suggesting not only the non‐toxic nature of the hydrogel but also its potential to stimulate L929 cell proliferation. We further supplemented the cytocompatibility test of hydrogel on human fibroblasts (HSF), including AO/EB live and dead cell staining and cytotoxicity assay. Experiments performed using the L929 cell model showed generally similar results to those using the HSF cell model (Figures S16,S17, Supporting Information), and there was no significant difference between N‐P/B/SH/[TA(siRNA)@BSA] and N‐P/B/SH/[TA(siRNA)@BSA]+bromophenol blue hydrogel groups.

After preparing TA(siRNA), TA(siRNA)@BSA, N‐P/B/SH, and N‐P/B/SH/[TA(siRNA)@BSA], we investigated their gene silencing efficiency (Figures S18,S19, Supporting Information). The results showed that TA(siRNA) as well as TA(siRNA)@BSA, and N‐P/B/SH/[TA(siRNA)@BSA] significantly increased the gene silencing efficiency compared to siRNA (gene silencing efficiency of ≈30%), suggesting that the gene silencing efficiency of siRNA was significantly increased in the N‐P/B/SH/[TA(siRNA)@BSA] hydrogel as well as TA (siRNA)@BSA nanogels possessed functional stability. In addition, N‐P/B/SH hydrogels also reduced MMP‐9 protein expression, which we attribute to the fact that nudibranch polysaccharides possess a good antioxidant effect, and this antioxidant mechanism produces a part of the efficacy in reducing MMP‐9 protein expression. Therefore, there may be a synergistic effect between this antioxidant effect and the gene silencing effect of siRNA. The increased gene silencing efficiency of TA (siRNA)@BSA nanogels compared to TA (siRNA) is due to the good biocompatibility of BSA as a coating as well as the transportation effect. Despite the possible synergistic effect of antioxidant action and gene silencing of siRNA in N‐P/B/SH/[TA(siRNA)@BSA] hydrogel, however, due to the slow‐release effect of the hydrogel, the concentration of TA(siRNA)@BSA in the culture broth is supposed to be lower than that of the TA(siRNA)@BSA nanogel added directly, thus resulting in a lower gene silencing efficiency of the N‐P/B/SH/[TA(siRNA)@BSA] hydrogel (78%) than the TA(siRNA)@BSA nanogel (93%).

Moreover, hemocompatibility testing outcomes (Figure S20, Supporting Information) showcased that the hemolysis rates of TA(siRNA)@BSA nanogel, N‐P/B/SH hydrogel, and N‐P/B/SH/[TA(siRNA)@BSA] hydrogel were all below 1%. These findings underscored the commendable cytocompatibility of N‐P/B/SH/[TA(siRNA)@BSA] hydrogel.

Impaired angiogenesis significantly retards the healing of diabetic wounds.^[^ ^19^ ^]^ The in vitro vascular formation assay, also known as the tubule formation assay, effectively mimics the process of angiogenesis and stands as a rapid, quantifiable, and classical approach to investigating angiogenesis in vitro.^[^ ^20^ ^]^ The pro‐angiogenic potential of the N‐P/B/SH/[TA(siRNA)@BSA] hydrogel was evaluated using the tubule formation assay. As depicted in Figure 4b, both the N‐P/B/SH/[TA(siRNA)@BSA] hydrogel and N‐P/B/SH hydrogel groups exhibited more conspicuous and densely packed tubular structures. Quantitative analysis (Figure 4f) revealed a notable increase in the number of branching points in both the N‐P/B/SH/[TA(siRNA)@BSA] hydrogel and N‐P/B/SH hydrogel groups compared to the control group, underscoring their capacity to facilitate blood vessel formation in endothelial cells.

The efficacy of the N‐P/B/SH/[TA(siRNA)@BSA] hydrogel on wound healing was evaluated using a cell scratch assay to mimic wound Figure 4c in vitro. Compared to the blank control, a notable enhancement in cell migration rate was observed, with a 56.7% increase in the N‐P/B/SH hydrogel group and a 97% increase in the N‐P/B/SH/[TA(siRNA)@BSA] hydrogel group (Figure 4e), indicating that the N‐P/B/SH/[TA(siRNA)@BSA] hydrogel facilitates cell migration and expedites wound healing. This phenomenon can be ascribed to the ability of paramylon and TA to enhance cell barrier function and metabolism, thereby fostering cell migration.^[^ ^21^ ^]^

The microenvironment of diabetic wounds sustains macrophages in a pro‐inflammatory phenotype, characterized by substantial secretion of inflammatory factors, culminating in chronic inflammation. Hydrogels endowed with anti‐inflammatory properties or immunomodulatory effects have the potential to induce macrophage phenotypic alterations, thus expediting wound healing.^[^ ^22^ ^]^

Treatment of macrophages with the N‐P/B/SH hydrogel and N‐P/B/SH/[TA(siRNA)@BSA] hydrogel prompted their differentiation toward the M2 phenotype. Lipopolysaccharide (LPS) was employed to induce the M1 phenotype of macrophages, characterized by robust expression of red fluorescently labeled CD86. Subsequent treatment with hydrogels demonstrated the ability of both N‐P/B/SH hydrogel and N‐P/B/SH/[TA(siRNA)@BSA] hydrogel to facilitate the transition of macrophages from the M1 phenotype to the M2 phenotype (evidenced by markedly stronger fluorescence of CD206 and diminished fluorescence intensity of CD86 compared to the LPS‐positive control). Furthermore, the impact of macrophage polarization was evaluated based on the expression levels of tumor necrosis factor‐α (TNF‐α) and interleukin‐10 (IL‐10), revealing a significant reduction in TNF‐α and a notable increase in IL‐10 following treatment with N‐P/B/SH hydrogel and N‐P/B/SH/[TA(siRNA)@BSA] hydrogel compared to the LPS group (see Figure S21, Supporting Information). Lipopolysaccharide triggers the activation of the NF‐κB signaling pathway.^[^ ^23^ ^]^ Tannic acid (TA) mitigates inflammation induced by oxidative stress and pro‐inflammatory cytokines by suppressing NF‐κB activation.^[^ ^24^ ^]^ Furthermore, both TA and paramylon possess antioxidant capabilities and can sequester iron, thus regulating oxidative stress within the inflammatory milieu.^[^ ^21^, ^25^ ^]^ The findings elucidate that the N‐P/B/SH/[TA(siRNA)@BSA] hydrogel effectively modulated macrophage secretion, facilitating a transition in the microenvironment from pro‐inflammatory to anti‐inflammatory states.

### 2.5 Biosensing Wound Monitoring Platform

Chronic wounds develop when the natural healing process is hindered by a multitude of underlying pathophysiological factors. Monitoring these diverse factors can furnish personalized diagnostic insights for wound management, yet prevailing sensing technologies rely on intricate laboratory analyses or monitor only a restricted set of wound parameters.^[^ ^26^ ^]^ Herein, we unveil a versatile biosensing platform facilitating comprehensive analyses of the wound microenvironment and real‐time monitoring of vital signs at the point of care.

**Figure 5:**
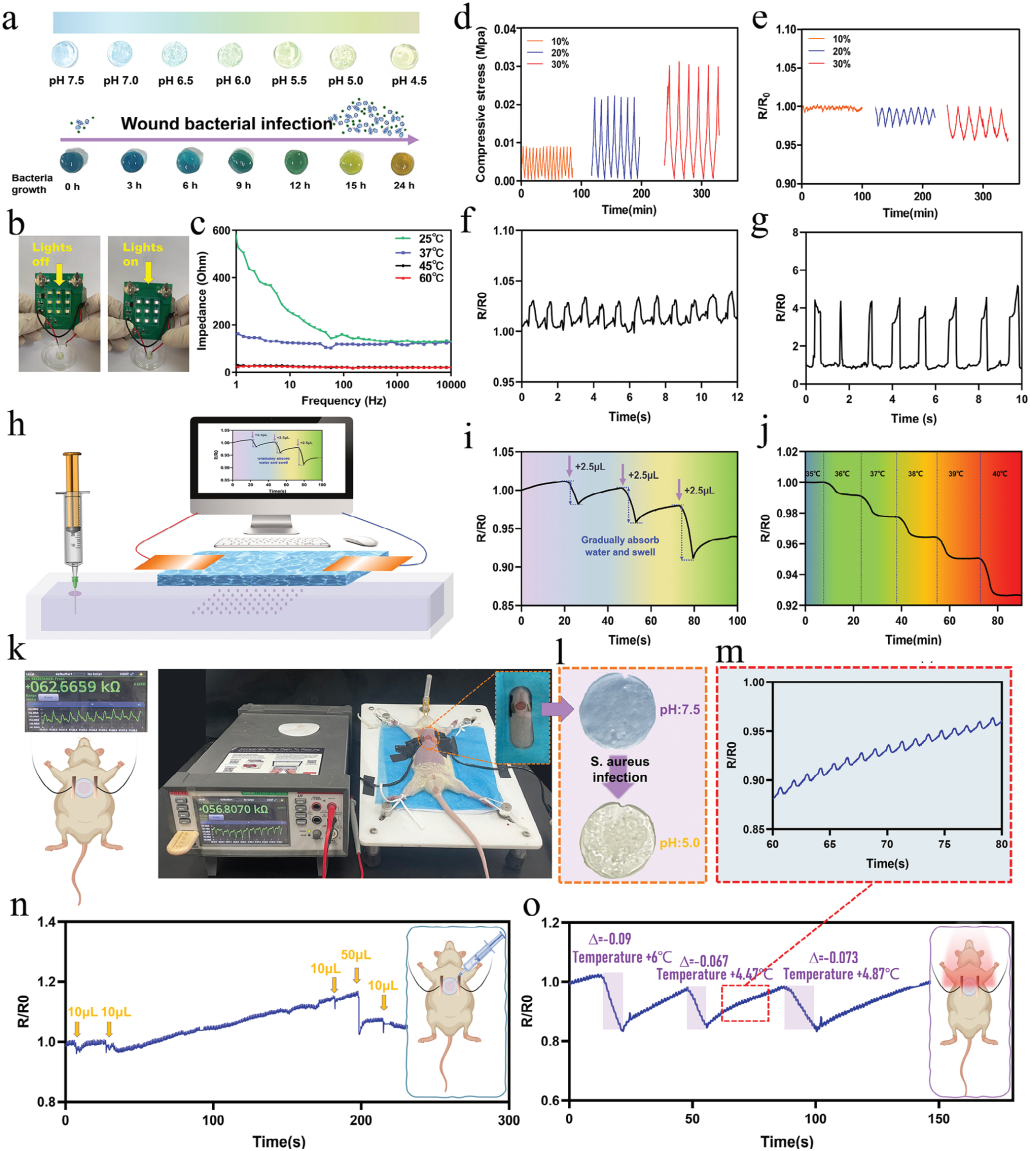
Conductive properties of hydrogels and wound signal monitoring: a) Detection of different pH and bacteria growth stages using the N‐P/B/SH/[TA(siRNA)@BSA] + bromophenol blue hydrogel; b) Conductivity of the N‐P/B/SH/[TA(siRNA)@BSA] hydrogel. Connection of the hydrogel completed a circuit to illuminate light‐emitting diodes (LEDs); c) Interfacial impedance of N‐P/B/SH/[TA(siRNA)@BSA] hydrogel on pig skin at different temperatures; d) Time‐cycles compression stress curve at 10, 20, and 30% strain; e) Relative resistance change of the sensors for different compression strain; Demonstration of real‐time human motion detection using a N‐P/B/SH/[TA(siRNA)@BSA] hydrogel strain sensor attached to human skin. Resistance‐time change curves for f) bending the index finger, and g) bending the knee; h) Schematic device of simulating wound exudate and monitoring of N‐P/B/SH/[TA(siRNA)@BSA] hydrogel sensor; i) The normalized relative resistance of the N‐P/B/SH/[TA(siRNA)@BSA] hydrogel sensor when the volume of exudate from 0 to 12.5 µL; j) The normalized relative resistance of N‐P/B/SH/[TA(siRNA)@BSA] hydrogel sensor was responsive to the temperature from 35 to 40 °C; k)Schematic diagram of bacterial infection wound monitoring in rats; l) Photographs of bacteria‐infected open wounds diagnosis in rat; Recorded resistance with time to monitor changes in m) heartbeats, n) wound exudate and o) temperature.

The real‐time visualization of bacterial infection detection is pivotal for the timely diagnosis and treatment of diabetic wound infections, which are often chronic and insidious. Under normal physiological conditions, the pH remains neutral; however, bacterial proliferation induces acidification at the infection site. Leveraging bromophenol blue‐loaded hydrogels enables continuous monitoring of infection status, predicated on the reversible alterations in molecular conformation induced by varying pH levels, thereby eliciting a corresponding change in hydrogel hue. As depicted in **Figure** **5**a, the transition of the N‐P/B/SH/[TA(siRNA)@BSA) hydrogel dressing from a slate‐blue hue to a pale yellowish‐green tint after incubation in PBS solutions across diverse pH ranges (7.0–4.5) for 1 h is indicative of its potential for real‐time visualization of bacterial infections. As shown in Figure 5a, the color changes of the hydrogel under co‐incubation with different densities of Staphylococcus aureus cultures in microenvironments at different growth stages were used to verify the pH changes during bacterial growth. It was found that the color changes were similar to those after incubation in solutions with different pH values. This means that once an infection occurs, the color of the hydrogel changes from grayish blue to light yellowish green to indicate the occurrence of infection. This capability holds significant promise, particularly for the surveillance of protracted chronic inflammatory wounds in diabetic individuals.

The N‐P/B/SH/[TA(siRNA)@BSA] hydrogel demonstrates ionic conductivity facilitated by borax, leading to the illumination of a connected light‐emitting diode (LED) wire. Moreover, it showcases a diverse array of sensing capabilities. The electrical conductivity of hydrogels is caused by the migration of borate ions in water. The reason that hydrogel degradation leads to an increase in electrical conductivity (Figure S22a, Supporting Information) may be due to the collapse of the gel network or a decrease in the crosslink density, which makes the resistance to the movement of borate ions weaker, and therefore the electrical conductivity increases. Moreover, the decrease in water content resulted in a decrease in conductivity when the hydrogel was left at 37 °C (Figure S22b, Supporting Information), indicating that in order to maintain the conductivity within the normal resistance of the skin, it is necessary to ensure a certain amount of water when using the hydrogel.

#### 2.5.1 Evaluation of Strain Sensing Capabilities

Throughout tissue regeneration, excessive deformation at the defect site caused by impact, abrasion, tearing, and stretching could precipitate histopathological changes, subcutaneous capillary rupture, local congestion, and edema.^[^ ^27^ ^]^ Hence, ongoing in situ surveillance of the wound site becomes imperative, offering real‐time alerts for excessive deformation and enabling prompt intervention for the patient's benefit. We explored alterations in resistance amid cyclic compression of the N‐P/B/SH/[TA(siRNA)@BSA] hydrogel employing a universal test compressor alongside a DM6500 multimeter (Figures 5d,e). The findings revealed that under 10% compressive strain, the average rate of change in ΔR/R_0_ stood at 1%, yielding a sensitivity (GF = (ΔR/R_0_)/ε) of 0.1; at 20% compressive strain, the average rate of change in ΔR/R_0_ reached 2.5%, corresponding to a GF of 0.25; meanwhile, at 30% compressive strain, the average change in ΔR/R_0_ was 3.8%, with a GF of 0.38.

These findings suggest a heightened sensitivity when juxtaposed with other investigations. S. Li et al. developed a stretchable, bonded, and conductive PVA/PAM/GA‐Zn^2+^ hydrogel‐based strain sensor, which exhibited excellent mechanical properties and high sensitivity (GF = 0.209)^[^ ^28^ ^]^; S. Wang et al. developed a conductive wood‐based composite hydrogel (CWH) with stable strain sensitivity (GF = 0.2805, *R* ^2^ = 0.998) capable of monitoring human joint flexion and capturing facial smiles^[^ ^29^ ^]^; M. Du et al. developed poly(vinyl alcohol) (PVA)‐stabilized eutectic gallium and indium (EGaIn)/polyacrylamide (PAAm)/polyacrylic acid (PAA)@FeCl3@polyprrole (PPy) ion‐conducting hydrogels, and the conductive composite hydrogels have good electrical conductivity and acceptable sensitivity (GF of 0.28).^[^ ^30^ ^]^ B. Yang reported a GF of 0.2 for PAM/Agar/LiCl hydrogels subjected to 50% deformation.^[^ ^31^ ^]^ It can be seen that the conductive hydrogel developed in this study (GF = 0.1–0.38, ΔR/R_0_ = 0.001–0.114, conductivity: 1 × 10^−3^ for this study) is at the better level of ionic conductive gels. The concept of bionics is that materials that are close to the body's electrical conductivity (electrical conductivity of human skin is in the range of 1 × 10^−5^–0.26 S m^−1^) are better able to promote tissue repair. The low‐strain sensing mechanism finds utility in detecting subtle bodily alterations, including those involving finger skin (with deformation under 20%), ACL (4% deformation), tendons (10% deformation), and spinal ligaments (deformation ranging from 10% to 40%). As depicted in Figures 5f,g, the N‐P/B/SH/[TA(siRNA)@BSA] hydrogel adeptly monitors the periodic angular shifts of fingers and knees at 90°, delivering synchronized, steadfast, and recurrent signals. Consequently, it holds promise for tracking significant bodily deformations and facilitating motion monitoring.

#### 2.5.2 Evaluation of Temperature Sensing Capability

Temperature, a crucial physiological parameter, serves as a pivotal indicator of the human body's state, wherein wound infection and inflammation often prompt a rise in body temperature. Initially, we scrutinized the AC impedance of the N‐P/B/SH/[TA(siRNA)@BSA] hydrogel across varying temperatures, as depicted in Figure 5c, unveiling a progressive decline in the hydrogel's AC resistance as the temperature ascends from 25 to 60 °C. This phenomenon stems from the temperature sensitivity inherent in the borate bonds of the N‐P/B/SH/[TA(siRNA)@BSA] hydrogel, wherein elevated temperatures trigger a partial rupture of these bonds, inducing a relaxation in the gel network, heightened ionic mobility, and consequent reduction in AC resistance. Typically, the skin's surface temperature ranges between 35 and 40 °C. We systematically elevated the temperature from 35 to 40 °C, observing that the N‐P/B/SH/[TA(siRNA)@BSA] hydrogel consistently retained notable temperature sensitivity. Illustrated in Figure S23 (Supporting Information), the standard curve for temperatures ranging from 35 to 40 °C unveils a quadratic correlation between temperature and R/R_0_ (with an *R* ^2^ value exceeding 0.99).

#### 2.5.3 Exudate Sensor

Factors elevating capillary permeability or triggering tissue edema (e.g., inflammation, bacterial infection) engender wound exudate. Its quantity may signal systemic issues like dehydration, hypovolemic shock, and microvascular lesions, or serve as a hallmark of ischemic ulcers, underscoring the significance of exudate monitoring. We reference a device^[^ ^32^ ^]^ that mimics wound exudate by expelling saline from a syringe, depicted in Figure 5h. Notably, Figure 5i illustrates the sensor's responsiveness to exudates ranging from 5 to 20 µL owing to ion augmentation within the exudate. This sensitivity stems from the hydrogel's commendable solubility, facilitating substantial water absorption and consequent hydrogel expansion, thus inducing a resistance alteration.

To further validate the multifunctional monitoring capabilities of the hydrogel, we conducted real‐time monitoring using a rat model with infected wounds. Application of the hydrogel to the wound induced a color change from the original blue (pH 7.5) to yellow (pH 4.5) (Figure 5l), signifying the presence of infection. This conspicuous alteration in hydrogel color underscores its potential for real‐time visualization of bacterial infections, particularly in the surveillance of chronic inflammatory wounds in diabetic patients. Furthermore, distinct voltage signal waveforms generated by pressure and temperature stimuli enable differentiation between temperature variations (Figure 5m), exudate levels (Figure 5n), and heart rate (Figure 5o), facilitating the analysis and quantification of exudate volume or body temperature fluctuations through changes in electrical resistance.

### 2.6 Promoting Wound Healing

**Figure 6:**
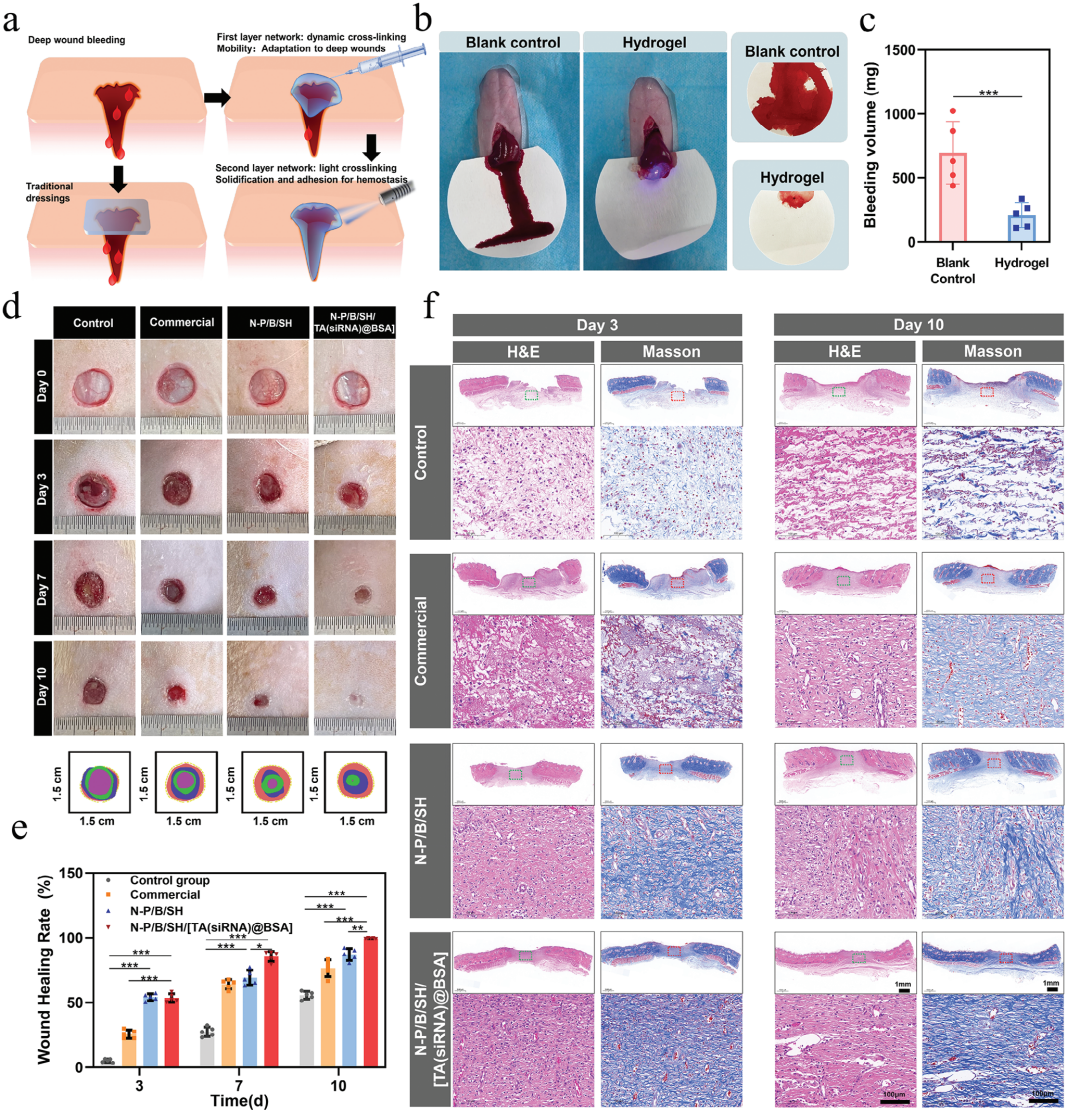
Hemostatic properties of hydrogels and promotion of diabetic chronic wound healing: a) Schematic diagram of the hemostatic mechanism of hydrogels; b) Bleeding conditions of the liver with or without the hydrogel; c) Hemostatic performance of the hydrogels; d) Full‐thickness skin defects in diabetic rats and the healing trajectory of the defects; e) The healing rates of the defects on days 3, 7, and 10; f) The low‐magnification (scale bar: 1 mm) and high‐magnification (scale bar: 100 µm) of H&E and Masson staining images of the defects on days 3 and 10; ^*^ *p* < 0.05, ^**^ *p* < 0.01, ^***^ *p* < 0.001, *n* = 5.

Hemostasis constitutes the inaugural phase of wound repair, prompting our scrutiny of the hydrogel's hemostatic prowess. As illustrated in **Figure** **6**a and N‐P/B/SH/[TA(siRNA)@BSA] hydrogel exhibited a marked reduction in bleeding. Within 30 s of irradiation, a second network of hydrogel preforms is rapidly formed, forming a solid gel that seals bleeding wounds. Unlike traditional wound dressings, which possess static morphologies, this hydrogel offers the flexibility to address bleeding in deeper wound sites.

We established a diabetic wound model and depicted the healing progression of each treatment cohort in Figure 6c. Notably, discernible white abscesses were evident in the control group by day 3, suggestive of potential infection and suppuration. The results showed that the number of colonies in the N‐P/B/SH/[TA(siRNA)@BSA] hydrogel group was much lower than that in the control group, and the inhibition rate of the bacteria could reach more than 93% compared with the control group (Figure S24, Supporting Information). Both the N‐P/B/SH hydrogel and the N‐P/B/SH/[TA(siRNA)@BSA] hydrogel expedited wound healing compared to the control and commercial dressing groups. Remarkably, the N‐P/B/SH/[TA(siRNA)@BSA] hydrogel cohort exhibited the smallest wound area, underscoring the superior efficacy of this synergistic intervention in fostering wound closure. By day 10, wounds in the N‐P/B/SH/[TA(siRNA)@BSA] hydrogel group achieved full closure, whereas those in the control group remained incompletely healed until day 18, implying a notable 44.4% reduction in wound repair duration facilitated by the N‐P/B/SH/[TA(siRNA)@BSA] hydrogel.

Diabetic wound tissue repair was evaluated via H&E and Masson staining. By day 3, substantial inflammatory cell infiltration was evident in the control and commercial groups, alongside multifocal lymphocytic infiltration and multinucleated giant cells. Conversely, the N‐P/B/SH hydrogel and N‐P/B/[TA(siRNA)] hydrogel groups exhibited diminished inflammatory infiltration, underscoring the efficacy of both hydrogels in mitigating inflammation. By day 10, the epidermal architecture of the N‐P/B/SH/[TA(siRNA)@BSA] hydrogel cohort had fully regenerated, featuring observable blood vessels and other tissue attachments in the dermal layer, thereby underscoring the hydrogel's efficacy in fostering wound closure. Masson staining revealed a notably deeper blue hue and heightened collagen deposition in both the N‐P/B/SH hydrogel and N‐P/B/SH/[TA(siRNA)@BSA] hydrogel groups compared to the control and commercial counterparts. Quantitative analysis (Figure S24, Supporting Information) demonstrated that by day 10 of treatment, the relative levels of collagen deposition in the N‐P/B/SH/[TA(siRNA)@BSA] hydrogel group were 2351% of those in the control group, 1742% of those in the commercial group, and 864% of those in the N‐P/B/SH hydrogel group, affirming the substantial tissue remodeling potential of the N‐P/B/SH/[TA(siRNA)@BSA] hydrogel.

Macrophages serve as pivotal orchestrators of immune responses and wound healing processes. To elucidate the mechanisms through which N‐P/B/SH/[TA(siRNA)@BSA] hydrogel modulates macrophage phenotype and mitigates inflammation, we performed immunofluorescence staining on tissues to delineate macrophage phenotypes. Typically, macrophages can undergo activation in response to the microenvironment, transitioning into distinct polarization states denoted as classically activated macrophages (M1 phenotype) and alternatively activated macrophages (M2 phenotype).^[^ ^33^ ^]^

**Figure 7:**
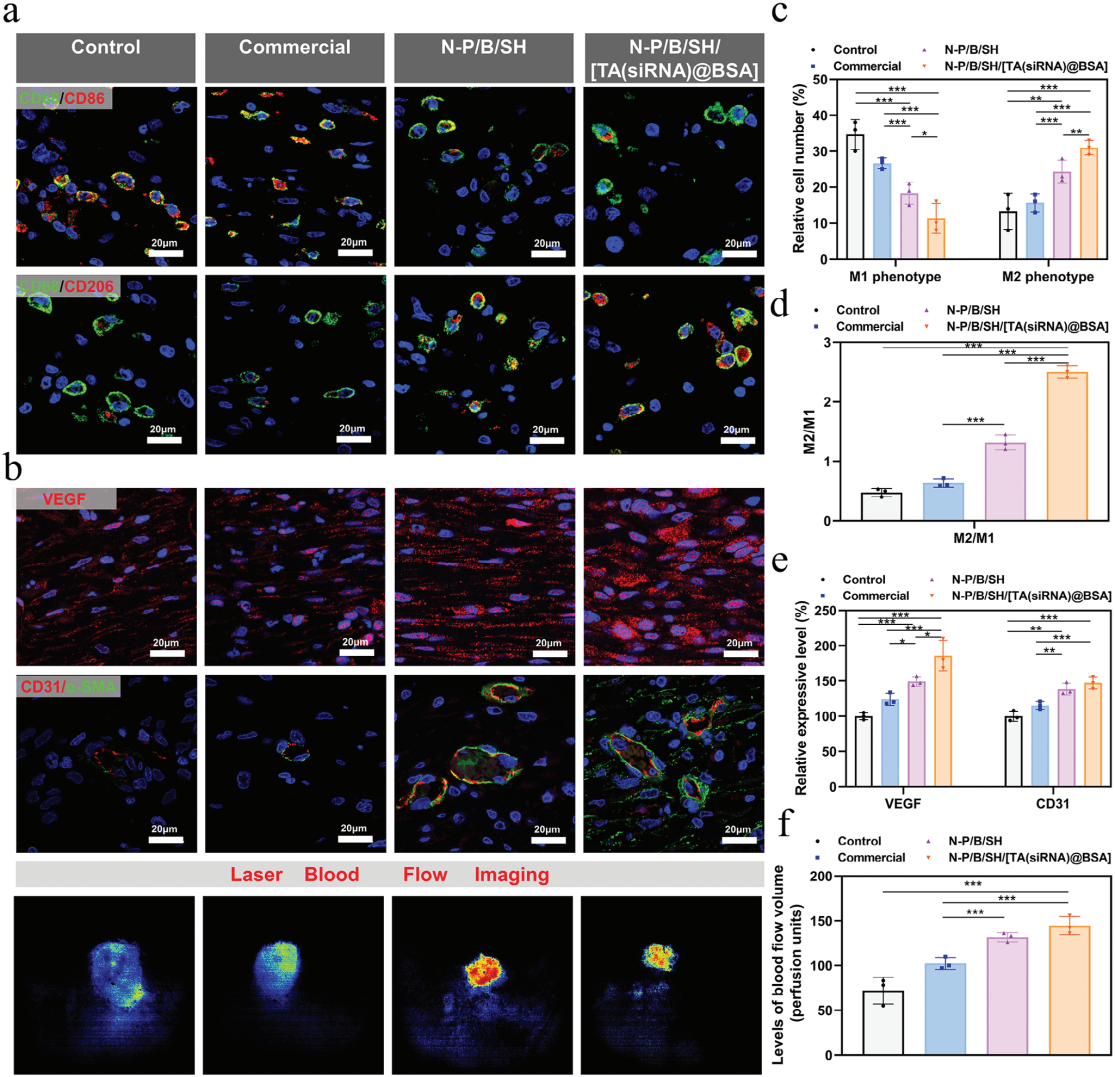
Immunomodulation, and vascularization: a) The immunofluorescent staining: CD86‐labeled M1 macrophages are indicated in red, and CD206‐labeled M2 macrophages are indicated in green, the blue color represents DAPI‐stained cell nucleus. The analyses were performed on day 7, and the scale bar represents a 20 µm scale; b) The immunofluorescent staining of the VEGF, and CD31 expressed and the laser speckle contrast imaging (LSCI) images of the defects collected on day 7; c) The population of CD86‐labeled M1 macrophages and that of CD206‐labeled M2 macrophages; d) The population ratios of M2 macrophages to M1 macrophages; e) The amounts of the VEGF, and CD31 expressed in the diabetic chronic wounds. The intensities of the fluorescent signals in the control groups were set to 100%; f) The perfusion indexes in different groups; ^*^ *p* < 0.05, ^**^ *p* < 0.01, ^***^ *p* < 0.001, *n* = 3.

**Figure** **7**a illustrates that the control and commercial groups exhibited a preponderance of M1‐type macrophages with scarce M2 representation. Conversely, both the N‐P/B/SH hydrogel and N‐P/B/SH/[TA(siRNA)@BSA] hydrogel assemblies manifested a discernible presence of M2‐type macrophages, notably, the N‐P/B/SH/[TA(siRNA)@BSA] hydrogel group exhibited the most pronounced abundance of M2‐type macrophages. The M2/M1 number ratio was computed, revealing a significantly higher proportion in the N‐P/B/SH/[TA(siRNA)@BSA] hydrogel group compared to others, indicative of its capacity to foster macrophage polarization. Notably, TA and paramylon exhibit potent anti‐inflammatory and antioxidant properties.^[^ ^21^, ^34^ ^]^ Moreover, the MMP‐9 silencing facilitated by TA‐siRNA nanogels contributes to inflammation reduction. The synergy of these factors amplifies the impact of N‐P/B/SH/[TA(siRNA)@BSA] hydrogel on macrophage M2 polarization.

Impaired vascularization commonly retards the healing process of diabetic wounds since the establishment of new blood vessels is imperative for oxygen exchange and nutrient transport within the wound milieu. We evaluated blood flow and the emergence of nascent vascular networks at the wound locus. As depicted in Figure 7b, the diabetic rat total cortical injury wound model's control and commercial groups manifested diminished blood flow by day 7, contrasting with the augmented blood flow observed in the N‐P/B/SH hydrogel and N‐P/B/SH/[TA(siRNA)@BSA] hydrogel group, with the latter exhibiting the most pronounced enhancement.

Vascular endothelial growth factor (VEGF), also known as vascular permeability factor (VPF), is a highly specific endothelial growth factor. VEGF orchestrates wound healing by instigating angiogenesis, augmenting vascular permeability, and expediting granulation tissue formation. VEGF is produced by many cell types, including vascular endothelial cells, tumor cells,^[^ ^35^ ^]^ macrophages,^[^ ^36^ ^]^ platelets,^[^ ^37^ ^]^ keratinocytes,^[^ ^38^ ^]^ and glomerular mesangial cells.^[^ ^39^ ^]^ From this study, VEGF produced in skin tissue may come from vascular endothelial cells, macrophages, or keratinocytes. CD31, also known as platelet‐endothelial cell adhesion molecule, is found mostly in the tight junctions between endothelial cells and on the surface of platelets,^[^ ^40^ ^]^ and to a lesser extent in neutrophils, monocytes, and some types of T cells.^[^ ^41^ ^]^ Endothelial cells are stained by CD31, as a marker for endothelial cells, and pericytes/smooth muscle cells are stained by α‐SMA, as a marker for pericytes/smooth muscle cells. Mature blood vessels consist of endothelial cells and pericytes/smooth muscle cells, stabilizing vascular structures and controlling their permeability. A mature blood vessel would be presented as a vessel of CD31(+) and α‐SMA (+).^[^ ^42^ ^]^ In this study, the size of the vessels as well as the number of vessels can be seen by double staining of CD31 and α‐SMA. The outcomes (depicted in Figure 7b) unveiled that the N‐P/B/SH/[TA(siRNA)@BSA] hydrogel cohort exhibited the highest VEGF and CD31 expression levels, coupled with a notable surge in blood vessel count compared to its counterparts. These findings underscore the potent neovascularization induction potential of the N‐P/B/SH/[TA(siRNA)@BSA] hydrogel.

Mechanisms of Vascular Promotion by N‐P/B/SH/[TA(siRNA)@BSA] Hydrogel: 1) Polarized M2 macrophages can secrete VEGF through exosomes, or influence other cells to produce VEGF through exosomes, or regulate VEGF production through the TGF pathway,^[^ ^36^ ^]^ fostering endothelial cell sprouting, thereby commanding the initiation of the angiogenic cascade and bolstering neovascularization. 2) In our antecedent investigation, we unveiled the potential of nudibranch polysaccharide to augment the HIF‐VEGF pathway, a catalyst for blood vessel regeneration. Consequently, N‐P/B/SH hydrogel exhibited a significant advantage in vascularization compared to both the untreated and commercial groups. 3) The TA(siRNA) nanogel encapsulated within N‐P/B/SH/[TA(siRNA)@BSA] hydrogel curtails MMP‐9 expression, ameliorating the inflammatory and oxidative stress milieu, thereby fostering vascular regeneration. Thus, N‐P/B/SH/[TA(siRNA)@BSA] hydrogel surpasses N‐P/B/SH hydrogel in fostering vascular regeneration.

**Figure 8:**
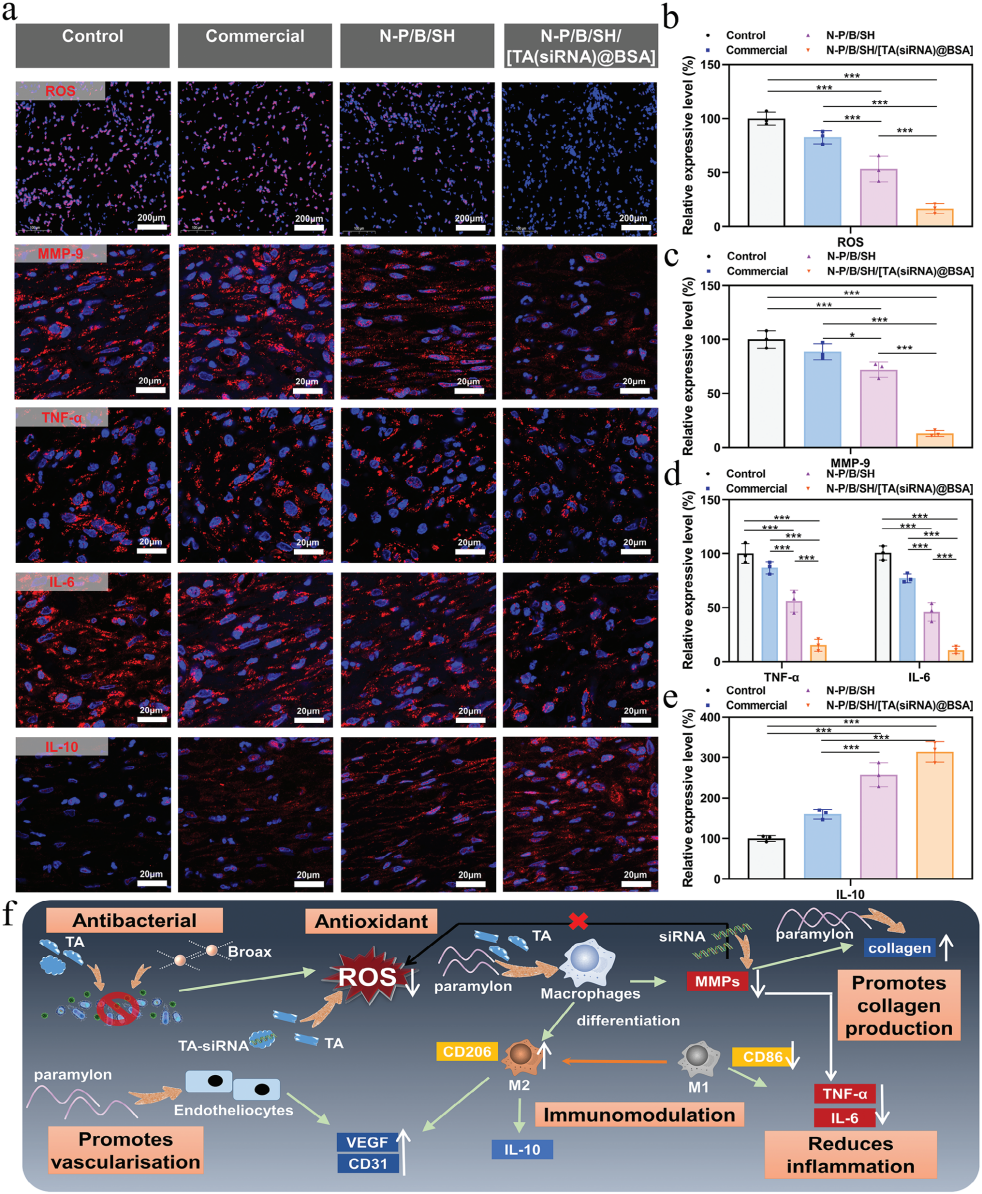
Oxidative stress and inflammation: a) The labeling of the ROS(200 µm scale) and the immunofluorescent staining of the MMP‐9, TNF‐α, IL‐10, and IL‐6 expressed(20 µm scale). The blue color represents the DAPI‐stained cell nucleus. The ROS was labeled with a DHE fluorescent probe (violet color); The quantitative analyses for the intensities of the fluorescent signals produced by b) ROS, c) MMP‐9, d) TNF‐α and IL‐6, e) IL‐10, The intensities of the fluorescent signals in diabetic chronic wounds in the control groups were set to 100%; f) The mechanism for the repair of diabetic wounds using N‐P/B/SH/[TA(siRNA)@BSA] hydrogel; ^*^ *p* < 0.05, ^***^ *p* < 0.001, *n* = 3.

The anti‐inflammatory and antioxidant mechanisms of the N‐P/B/SH/[TA(siRNA)@BSA] hydrogel for wound healing were subjected to further scrutiny. Functioning as a carrier, the TA‐siRNA nanogel transports MMP‐9 siRNA into cells, thereby attenuating cellular MMP‐9 expression. Immunofluorescence staining of MMP‐9 in diabetic rat wound tissues was conducted, with results depicted in **Figures** **8**a,b. The control group exhibited pronounced MMP‐9 expression, whereas solely the N‐P/B/SH/[TA(siRNA)@BSA] hydrogel group manifested the most substantial reduction in MMP‐9 expression, underscoring the effective silencing of the MMP‐9 gene by the TA‐siRNA nanogel.

Excessive accumulation of reactive oxygen species (ROS) in wounds not only triggers a robust inflammatory response but also impedes macrophage function, hindering tissue regeneration. In vitro experiments have confirmed the ROS‐scavenging efficacy of N‐P/B/SH/[TA(siRNA)@BSA] hydrogel. Furthermore, ROS levels were assessed in skin wound tissues, as depicted in Figure 8b. Dihydroethidium (DHE), also known as hvdroethidine, is one of the most commonly used probes for fluorescence detection of superoxide cations for the detection of ROS.DHE itself is a cell membrane‐permeable blue probe (λEXAEm: 370/420 nm), which, once inside the cell, reacts with superoxide cations to produce 2‐hvdroxvethidium, 2‐hvdroxyethidium inserts into nucleic acids and produces red fluorescence (A ExA Em: 535/610 nm) that can be detected by fluorescence microscopy, flow cytometry, or other suitable instruments. Since it fluoresces red upon insertion of nucleic acids, it overlaps with DAPI (blue), which labels the nucleus of the cell, and thus displays a purple color. Both the control and commercial dressing groups exhibited elevated ROS levels, whereas significant reductions were observed in ROS content with both N‐P/B/SH hydrogel and N‐P/B/SH/[TA(siRNA)@BSA] hydrogel treatments, attributed to the antioxidant properties of paramylon and TA. The results from this experiment (Figure 8a) indicate that N‐P/B/SH/[TA(siRNA)@BSA] hydrogel can promote intracellular antioxidant effects. And how they induce the intracellular antioxidant mechanism needs to be investigated in detail and depth next. Additionally, the reduction in MMP‐9 levels may contribute to the amelioration of the oxidative stress microenvironment.

Investigation into the inflammatory microenvironment of the wound unveiled the presence of pro‐inflammatory factors IL‐6 and TNF‐α, alongside the anti‐inflammatory factor IL‐10, as depicted in Figure 8a. Tissues from the control and commercial groups exhibited heightened secretion of IL‐6 and TNF‐α, coupled with diminished expression of IL‐10, signifying a pro‐inflammatory milieu. Both N‐P/B/SH hydrogel and N‐P/B/SH/[TA(siRNA)@BSA] hydrogel mitigated IL‐6 and TNF‐α levels while elevating IL‐10 expression to varying extents, with N‐P/B/SH/[TA(siRNA)@BSA] hydrogel demonstrating the most pronounced effect. These findings underscore the ability of N‐P/B/SH/[TA(siRNA)@BSA] hydrogel to foster a transition from a pro‐ to an anti‐inflammatory state within the tissue microenvironment, thereby mitigating inflammation and fostering wound healing.

To evaluate the in vivo safety profile of N‐P/B/SH/[TA(siRNA)@BSA] hydrogel, histological examination using H&E staining was conducted on vital organs (heart, liver, kidney, lungs, and spleen) of diabetic rats at day 7 post‐total cortical injury, as depicted in Figure S26 (Supporting Information). Notably, no discernible organ damage was observed across all groups, thus validating the safety profile of N‐P/B/SH/[TA(siRNA)@BSA] hydrogel as a viable wound dressing.

## 3 Conclusion

In tackling the complex challenges posed by conditions like chronic and infected wounds, this study pioneers the development of a nanocomposite hydrogel designed for the real‐time monitoring of wound dynamics and holistic therapy. Besides tracking pH, temperature, exudate variations, and peristalsis throughout the healing process, this hydrogel exhibits multifaceted functionality encompassing hemostatic, antibacterial, anti‐inflammatory, and antioxidant properties. Moreover, it orchestrates macrophage polarization and fosters vascular tissue regeneration. Ultimately, the platform envisioned herein amalgamates pathological monitoring with comprehensive therapeutic interventions, heralding promising horizons in biomedical endeavors.

## 4 Experimental Section

### Materials

Paramylon (Mw: 190 kDa, 98%) was provided by Yunnan Baoshan Zeyuan algal health technology Co., Ltd (Baoshan, China), Tannins, norbornene, bis‐terminal sulfhydryl PEG, lithium phenyl‐2,4,6‐trimethylbenzoylphosphite (LAP), BSA were purchased from Macklin Inc. The siRNA was purchased from Genepharma. Other reagents were used as received without further processing or purification.

### Preparation of TA‐siRNA@BSA Nanogels

1 mL (2 OD mL^−1^) of siRNA solution was placed under an ice‐water bath on a magnetic stirrer at a rate of 100 rpm, and 1 mL of TA (0.2%) solution was added slowly dropwise over 3 min for a 10 min reaction, and the resulting TA‐siRNA nanogel was purified by passing through a 100 kDa molecular weight cutoff centrifugal filtration device (Amicon Ultra‐ 4 Ultracel‐100K, Millipore) purified to remove excess unreacted reagents, and then RNAase‐free PBS buffer was added to 1 mL. 1 mL of BSA (0.5%) was added in PBS buffer to 1 mL of TA‐siRNA nanogel and mixed at room temperature for 2 h. The sample was purified to remove excess unreacted reagents to obtain BSA‐modified siRNA and TA self‐assembled TA‐siRNA@BSA nanogel.

### Characterization of Properties of Nanogels—Basic Physical and Chemical Properties and Morphology

The morphology of TA‐siRNA@BSA nanogel was observed using TEM. Particle size distribution, zeta potential, and gel fractions were examined. The details are described in Supporting Information.

### Characterization of Properties of Nanogels—Cell Internalization

Macrophages (Raw264) were used to study the cellular internalization of nanogels. The experimental groupings were siRNA and TA‐siRNA@BSA nanogel (100 µL/well, siRNA group: siRNA solution with a concentration of 2 OD mL^−1^, TA‐siRNA@BSA nanogel group: TA‐siRNA@BSA nanogel stock solution). The details are described in Supporting Information. Note that TA‐siRNA@BSA nanogel should be prepared under aseptic conditions, and the siRNA used for the preparation was green fluorescence‐labeled 5′FAM‐siRNA.

### Characterization of Properties of Nanogels—Gene Silencing

LPS solution was added to induce a pro‐inflammatory phenotype in macrophages (Raw264) (LPS concentration of 1 µg mL^−1^). The experimental groupings were the Control group (normal culture medium, without LPS to induce cellular inflammation), LPS model group (with the addition of LPS to induce cellular inflammation), siRNA (MMP‐9) group, and TA‐siRNA (MMP‐9) nanogel group. The cell crawls were stained with MMP‐9 immunofluorescence staining. Photographs were quantitatively analyzed by Image J software for fluorescence and gene silencing efficiency was calculated. The details are described in Supporting Information. Note the siRNA used for the preparation was green fluorescence‐labeled 5′FAM‐siRNA.

### Modification of Paramylon

The grafting of norbornene with paramylon (N‐P) is described in Supporting Information. The N‐P was detected by Fourier infrared spectroscopy (FT–IR) and thermogravimetric analyzer.

### Preparation of Hydrogel

0.2 g of borax was added to 8 mL of deionized water and the sample was mixed well until all dissolved. Then, 1 g of modified paramylon (N‐P) was added and made it completely dissolved, then 1 mL of TA‐siRNA@BSA nanogel stock solution was added, and 1 mL of cross‐linking agent initiator mixture (1 mL contained 1% bis‐terminal sulfhydryl PEG and 0.1% LAP), which was mixed completely and then subjected to blue light irradiation for 1 min, N‐P/B/SH/[TA(siRNA)@BSA] hydrogels could be formed. The N‐P/B/SH hydrogel was prepared by replacing 1 mL of TA(siRNA)@BSA nanogel stock solution in the above step with 1 mL of deionized water. N‐P/B/[TA(siRNA)@BSA] hydrogels were prepared by replacing 1 mL of the crosslinker initiator mixture solution in the above step with 1 mL of water. N‐P/B/SH/[TA(siRNA)@BSA] + bromophenol blue hydrogel was prepared by adding 100 µL of a 1% aqueous solution of bromophenol blue when dissolving the paramylon in the above step.

### Characterization of Properties of Hydrogels

Characterization of the basic properties of the hydrogel included: cryo‐scanning electron microscope observation of the morphology, Fourier transform infrared spectroscopy, thermogravimetric analysis, transmittance, swelling, mechanical properties, tissue adhesion, the release of siRNA, the bovine serum albumin adsorption.^[^ ^43^ ^]^ The details are described in Supporting Information.

### Cytology Experiment—In Vitro Cytotoxicity

The cytotoxicity of hydrogels was determined by MTT assay. The cell lines included L929 and HSF. The details are described in Supporting Information.

### Cytology Experiment—Live/Dead (L/D) Cell Staining Analysis

The viability of cells on the tested materials was evaluated using Live/Dead cell staining and fluorescence microscopy. The cell lines included L929 and HSF. The tested hydrogels included N‐P/B/SH hydrogel and N‐P/B/SH/[TA(siRNA)@BSA] hydrogel. The details are described in Supporting Information.

### Cytology Experiment—Cell Migration Assay

Cell migration experiments simulated wound healing in vitro. The cell line was L929. The experiments were divided into two groups: N‐P/B/SH hydrogel group and N‐P/B/SH/[TA(siRNA)@BSA] hydrogel group. The details are described in Supporting Information.

### Cytology Experiment—Tube Formation Experiment

Tube formation experiment to assess the pro‐angiogenic capacity of hydrogels. The cell line was HUVEC. The experiments were divided into two groups: N‐P/B/SH hydrogel group and N‐P/B/SH/[TA(siRNA)@BSA] hydrogel group. The details are described in Supporting Information.

### Cytology Experiment—Macrophage Polarization

Macrophage polarization experiments were conducted using mouse monocyte macrophage leukemia cells (RAW264.7). The experiments were grouped into control group (normal culture medium), lipopolysaccharide (LPS) group (concentration of 10 ng mL^−1^ of LPS, positive control), N‐P/B/SH hydrogel group (concentration of 50 mg mL^−1^ of paramylon extract), and LPS + N‐P/B/SH hydrogel group (concentrations the same as the LPS group and N‐P/B/SH hydrogel group). N‐P/B/SH/[TA(siRNA)@BSA] hydrogel group (concentration of 50 mg mL^−1^ of N‐P/B/SH/[TA(siRNA) extract), and LPS + N‐P/B/SH/[TA(siRNA)@BSA] hydrogel group (concentrations the same as the LPS group and N‐P/B/SH/[TA(siRNA)@BSA] hydrogel group). The RAW264.7 cells were inoculated on sterile coverslips and cultured for 24 h, and the supernatant was carefully removed, and the liquid from each group was added for another 24 h. Cell crawls were washed with PBS, fixed with 4% paraformaldehyde, subjected to immunofluorescence staining (CD206 (60143‐1, Proteintech), INOS (GB11119, Servicebio), and then observed under a laser confocal microscope (Nikon‐A1, Japan). The levels of inflammatory cytokines (TNF‐α and IL‐10) secreted by cultured RAW264.7 cells were determined using enzyme‐linked immunosorbent assay (ELISA) kits (Shanghai Enzyme‐Linked Biotechnology Co., Ltd., Shanghai, China).

### Antibacterial Test

The hydrogel's antibacterial activity toward *E*. *coli* and *Staphylococcus aureus* was evaluated. The hydrogel was prepared under aseptic conditions. Ten milliliters of bacterial suspension [10^6^ colony‐forming units CFU mL^−1^] was added to a sterilized lysogeny broth (LB) medium. One milliliter of hydrogel was added to the inoculated medium and allowed to sit for 24 h; the blank group was not added with the hydrogel. Afterward, the mixture was diluted by 10^6^ and spread on an LB solid medium. After incubation at 37 °C for 20 h, the number of colonies on the agar plates was counted.

To further investigate the antibacterial ability of the hydrogels, *E. coli* and *S. aureus* were inoculated in a liquid medium and co‐cultured with the hydrogels, and the optical density (OD600) of the bacterial solution was measured versus time.

The morphology of bacteria after different treatments was observed by SEM. The membrane structure of the bacteria was detected by TEM. The details are described in Supporting Information.

### Antioxidant Experiment

The antioxidant activity assay included an in vitro free radical‐scavenging assay (superoxide radical ^•^O_2_^−^, hydroxyl radical ^•^OH), DPPH scavenging assay, and ABTS radical cation (ABTS^·+^) scavenging assay. Details of experiment methods are available in the Supporting Information.

### Conductivity and Wound Monitoring

The AC resistance was measured at different temperatures (25, 37, 45, 60 °C) using an electrochemical workstation. Hydrogels were prepared as specific rectangles (1.5 cm long, 1 cm wide, 0.3 cm thick), connected to platinum sheet electrodes, and connected to the electrochemical workstation.

### Conductivity and Wound Monitoring—pH Monitoring

Hydrogel color changed responsive to pH Value and Bacterial Infections. In this test, the N‐P/B/SH/[TA(siRNA)@BSA]+ bromophenol blue hydrogel was punched into discs with a diameter of 1 cm and placed in each hole of the 24‐well plate. Then, 2 mL PBS buffer solution with different pH values (4.5, 5.0, 5.5, 6.0, 6.5, and 7.0) was gently added to the plate. After 2 h incubation, the hydrogels were photographed by a camera. The hydrogel was punched into discs with a diameter of 1 cm. Then, a strain of *S. aureus* was cultured in trypticase soy broth in a 24‐well plate for different times (0, 2, 4, 6, 8, and 10 h). Then, the nutrient broth mediums after centrifugation to remove bacteria were incubated with the hydrogel for 2 h at room temperature before recording the color changes of the hydrogels.^[^ ^44^ ^]^

### Conductivity and Wound Monitoring—Pressure‐Strain Monitoring of Motion Signals

For the cyclic compressive mechanical tests, the hydrogel was compressed with a strain of 10, 20, and 30%. The compression tests were performed at a rate of 10 mm min^−1^, and the modulus data were automatically calculated by the INSTRON 5565 instrument. To record the resistance changes of the N‐P/B/SH/[TA(siRNA)@BSA] hydrogel during compression deformation, the ends of the conductive N‐P/B/SH/[TA(siRNA)@BSA] hydrogels were attached to copper sheet electrodes to interface with a digital touch screen multimeter (Tektronix DMM6500). The resistance variations were recorded in real time at a time resolution of 0.5 s. The gauge factor (GF) was calculated using the following equation: *GF*  = Δ*R*/ε  =  (*R* − *R*_0_)/ε *R*_0_ (*R* was the resistance, *R_0_* was the initial resistance and *ɛ* was the strain).

A digital touchscreen multimeter (Tektronix DMM6500) was used to test the sensing performance of the hydrogel. To monitor human movement, conductive hydrogels were applied to the knee and finger areas and connected to a Tektronix DMM6500 with a copper wire. The Tektronix DMM6500 recorded changes in resistance with a time resolution of 0.5 s for simultaneous monitoring. The change in normalized relative resistance (or normalized relative capacitance) was calculated using the equation R/R_0_ (%).

### Conductivity and Wound Monitoring—Temperature Monitoring

Sensitivity of the temperature sensors. All evaluations of the temperature sensor were performed by putting the wound dressing in a thermostat with a temperature range from 35 to 45 °C.

### Conductivity and Wound Monitoring—Wound Exudate Monitoring

To simulate the absorption of wound exudates, hydrogels were put on a plate with small pores, and exudates penetrated the hydrogel through these pores, which finally caused signal changes of resistance. R/R_0_ represents the change in relative resistance to the initial value, R_0_ represents the initial resistance, and R represents real‐time resistance.

### Conductivity and Wound Monitoring—Animal Wound Monitoring

All experiments were performed in compliance with laws and institutional guidelines related to animal ethics, and conducted with the approval of the Institute Animal Ethics Committee of Northwest University (NWU‐AWC‐20220904M).

SD rats were used for pH, heartbeat, wound exudate, and temperature monitoring. After anesthesia, hair was removed from the abdomen of the rats and an infected wound (a circle with a diameter of 8 mm) was created, and 100 µL of Staphylococcus aureus bacterial suspension (bacterial concentration of 10^8^ units mL^−1^) was added dropwise, and a conductive hydrogel was placed on their abdomen after 30 min, with copper tabs on both sides of the hydrogel and wires connecting it to a multichannel touch‐screen digital meter (Tektronix DMM6500). To simulate exudation, a small amount of saline was dropped on the hydrogel. To simulate a rise in body temperature, an electric heater was placed above the rat wound. The pH change was determined by the color change of the hydrogel. Heartbeat, wound exudate, and temperature were monitored by the electrical resistance signal of the gel.

### Rat Liver Hemostasis

A rat hemorrhaging liver model was used to evaluate the in vivo hemostatic ability of the hydrogels. The liver of the rat was exposed after anesthesia. A filter paper was placed beneath the liver and sheared to bleed with surgical scissors. Immediately, 1 mL of the hydrogel was injected onto the surface of the bleeding site as a hemostatic agent and irradiated with blue light for 1 min, and the control group was left untreated. The extent of liver bleeding was observed, and the hemostatic properties of the hydrogel were judged by detecting the quantity of blood on the filter paper.

### Diabetic Wound Repair—Diabetic Rat Modeling

Rats whose blood glucose levels were greater than or equal to 16.7 mmol L^−1^ after being intraperitoneally injected with a 1% citrate‐buffered streptozotocin solution were selected as diabetic rats.

### Diabetic Wound Repair—Grouping and Wound Treatment

The experiment included a control group, a commercial dressing group, a N‐P/B/SH hydrogel group, and a N‐P/B/SH/[TA(siRNA)@BSA] hydrogel group. SD rats were anesthetized for dorsal debridement, and full‐thickness skin wounds (8 mm in diameter) were cut in the back of each rat using a punch biopsy after alcohol disinfection. Hydrogel group treatment: the hydrogel was applied to the skin wound, then covered and fixed with gauze and polyurethane film. Control group treatment: gauze and polyurethane film only. The hydrogels (N‐P/B/SH gel and N‐P/B/SH/[TA(siRNA)@BSA] gel) were changed daily to ensure moisture retention in the hydrogel and a moist environment for the wound. The commercial dressing is DuoDERM.

### Diabetic Wound Repair—Histological staining

The tissues were embedded in paraffin and sectioned before being subjected to H&E, Masson DHE, and immunofluorescent staining. Immunofluorescence single staining: VEGF (bs‐1313R, Bioss), CD86 (ab270719, abcam), CD68(GB113109, Servicebio), CD206 (60143‐1, Proteintech), TNF‐α (bs‐10802R, Bioss), MMP‐9 (bs‐4593R, Bioss), IL‐6 (bs‐0782R, Bioss) and IL‐10 (bs‐6761R, Bioss). Immunofluorescence double staining: α‐SMA (bsm‐33188 M, Bioss)/CD31 (GB11063‐2, Servicebio). The cell nuclei of the tissues were labeled with DAPI, and the photographs were analyzed for intensities of fluorescent signals using ImageJ software. The intensities of the fluorescent signals in the control groups were set to 100%. Laser Speckle Contrast Imaging (RFLSI Pro) was used to examine blood perfusion.

### Statistical Analysis

At least three independent replications for each data. Data are presented as means ± standard deviations. Statistical analysis was performed using SPSS software. One‐way or two‐way ANOVA was used to analyze significant differences at different thresholds: ^*^ *p* < 0.05, ^**^ *p* < 0.01, and ^***^ *p* < 0.001.

## Conflict of Interest

The authors declare no conflict of interest.

## Supporting information



Supporting Information

## Data Availability

Research data are not shared.
