# Supplementary material for: Nanocomposite Hydrogel for Real‐Time Wound Status Monitoring and Comprehensive Treatment
Source: Adv Sci (Weinh). 2024 Sep 13;11(42):2405924. doi: 10.1002/advs.202405924 (PMC11558094; doi:10.1002/advs.202405924)
Supplement: Supplementary file 1 — Supporting Information [file ADVS-11-2405924-s001.pdf]

ADVANCED SCIENCE
Open Access

Supporting Information

for *Adv. Sci.*, DOI 10.1002/advs.202405924

Nanocomposite Hydrogel for Real-Time Wound Status Monitoring and Comprehensive Treatment

*Huan Lei, Xueqing Yu and Daidi Fan**

Supporting Information

Nanocomposite Hydrogel for Real-Time Wound Status Monitoring and Comprehensive Treatment

Huan Lei[a, b, c,1], Xueqing Yu[a, b, c,1], Daidi Fan[a, b, c,*]

[a] Engineering Research Center of Western Resource Innovation Medicine Green Manufacturing, Ministry of Education, School of Chemical Engineering, Northwest University, Xi'an, 710069, China

[b] Shaanxi Key Laboratory of Degradable Biomedical Materials and Shaanxi R&D Center of Biomaterials and Fermentation Engineering, School of Chemical Engineering, Northwest University, Xi'an, 710069, China

[c] Biotech. & Biomed. Research Institute, Northwest University, Xi'an, 710069, China

[*]Corresponding Author

E-mail: fandaidi@nwu.edu.cn

Tel: 086-029-88305118; Fax: 086-029-88322585

ORCID ID

Daidi Fan ID (0000-0001-9798-1674)

Results

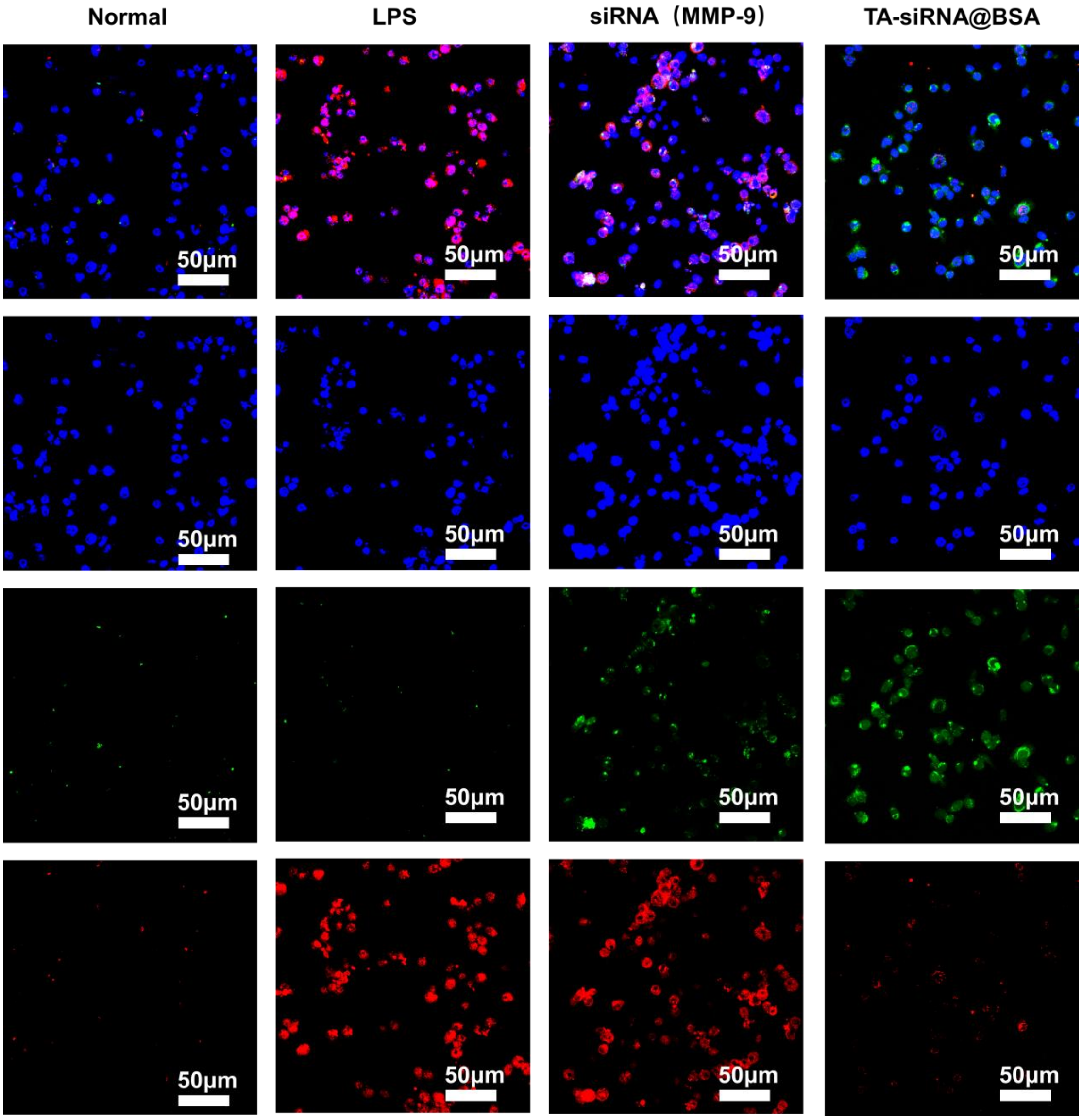

Figure S1. Efficiency of TA-siRNA @BSA on *MMP-9* gene silencing: split-channel plot of immunofluorescence staining. siRNA was labelled with 5-FAM, MMP-9 expression was assessed using immunofluorescence staining. Cell nuclei were labelled with DAPI (blue). Scale bar is 20 μm.

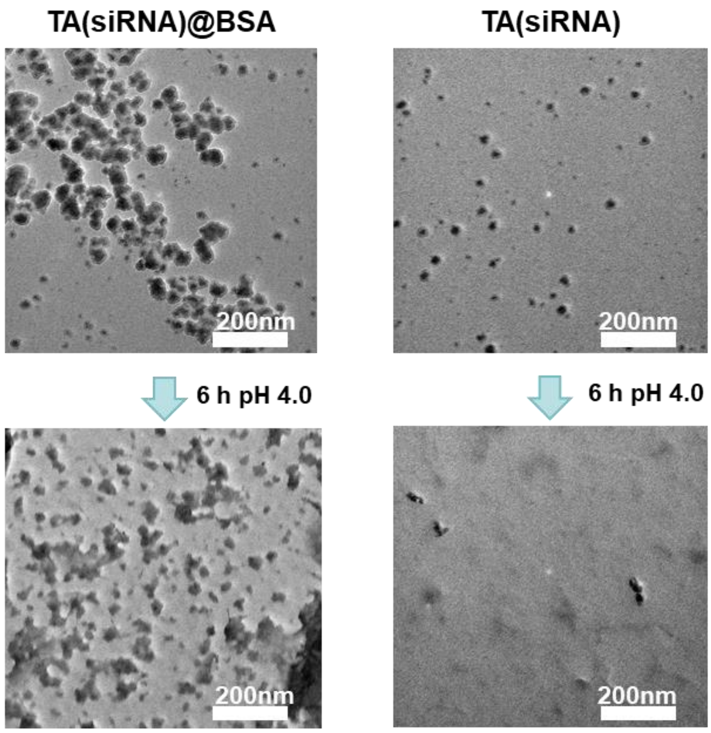

Figure S2. Transmission electron microscopy of TA(siRNA)@BSA nanogel and TA(siRNA) nanogel after 6h treatment in acidic environment

We observing the morphology of the nanogel by TEM after 6 h in an acidic environment, as shown in the figure S2, at 6 h in an acidic environment, the TA(siRNA)@BSA nanogel showed degradation, but part of the gel could still be observed, whereas the TA(siRNA) was almost completely degraded, suggesting that the BSA enhanced its stability.

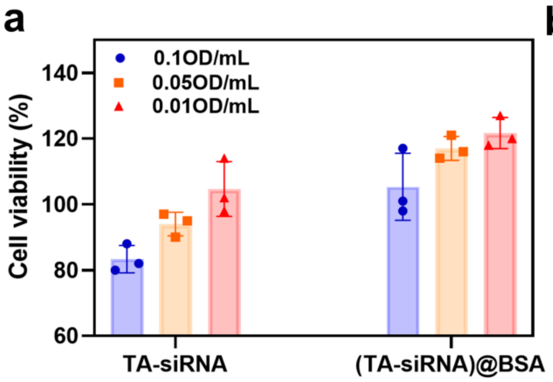

b

Table ISO10993.12-2005

Relative cell viability/%	Toxicity grade
≥100	0
75-99	1
50-74	2
25-49	3
1-44	4
0	5

Figure S3. a) The effect of TA(siRNA)@BSA nanogel, TA(siRNA) nanogel on the cell viability of human fibroblasts at different concentrations; b) International standard: classification of toxicity classes of biomaterials

The cell survival rate was 83% at a TA-siRNA nanogel concentration of 0.1 OD/mL. According to the national standard ISO10993.12-2005, the cytological toxicity of the materials were all class 1, which is in accordance with the medical standard. However, TA(siRNA)@BSA nanogel at a concentration of 0.1OD/mL had a cell survival rate of >100% and a cytotoxicity grade of 0. Moreover, as the concentration decreased, the TA(siRNA)@BSA nanogel promoted cell proliferation more significantly. This is partly due to the TA is an acidic substance, which causes a change in the pH of the cell culture solution at higher concentrations, and thus a lower cell survival rate occurs at high concentrations. In addition, the BSA coating improves the stability of the nanogel and has a slow-release effect, and the release of TA is delayed, so the cell survival rate is increased. And since BSA is a nutrient, it helps in cell proliferation. Therefore, the slow-release effect provided by the BSA coating (supporting Figure S3), as well as the cell-proliferative effect of BSA can alleviate the damage of TA on cells.

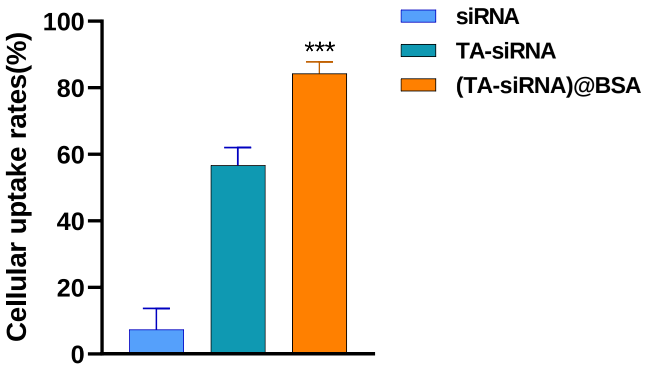

Figure S4. Cellular uptake rate of siRNA, TA-siRNA, (TA- siRNA)@BSA (***: $p < 0.001$, $n = 5$)

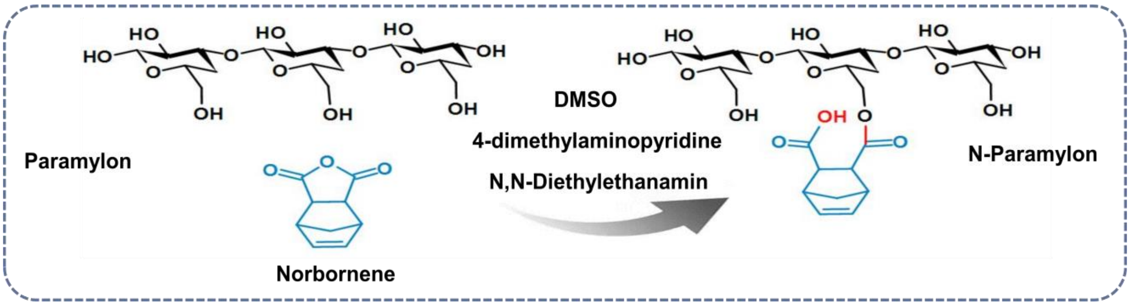

Figure S5. Schematic diagram of modified paramylon: grafted norbornene

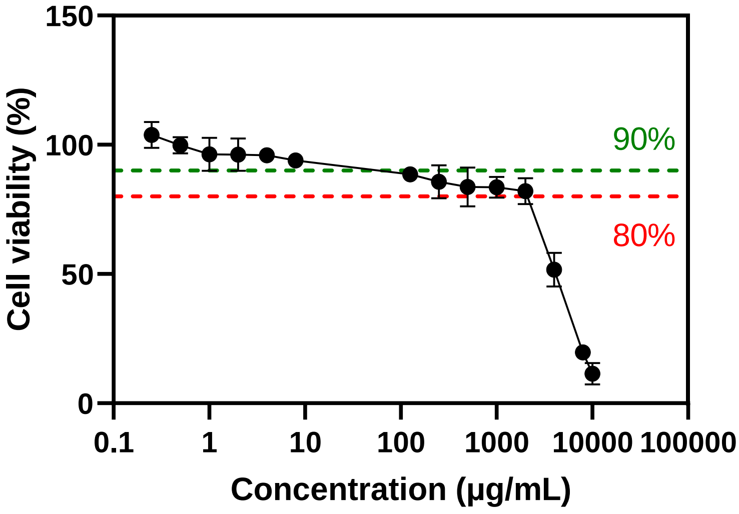

Figure S6. Cytotoxicity of different concentrations of norbornene

The cell survival rate of norbornene was 90% at a concentration of 8 μg/mL or less, 90%-80% at 125-2000 μg/mL, and a large number of cells died at a concentration greater than 2000 μg/mL. According to the cytotoxicity standard of biomedical materials stipulated by the national standard, the concentration of norbornene should be controlled below 2mg/mL.

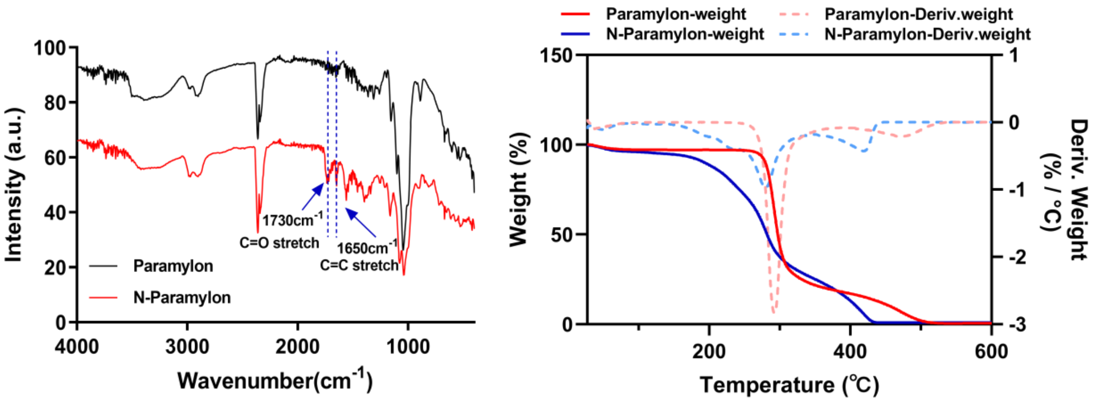

Figure S7. Fourier infrared spectroscopy (a) and thermogravimetric curves (b) of modified paramylon.

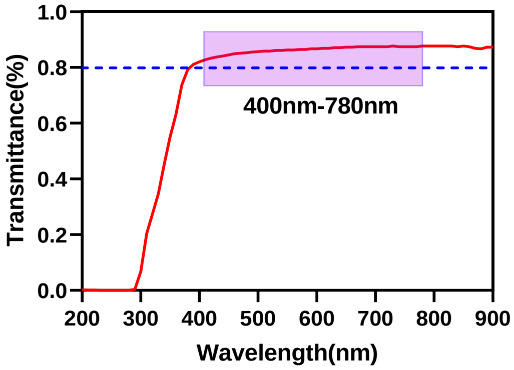

Figure S8. UV-Vis transmittance spectra of the N-P/B/SH/[TA(siRNA)@BSA] hydrogel.

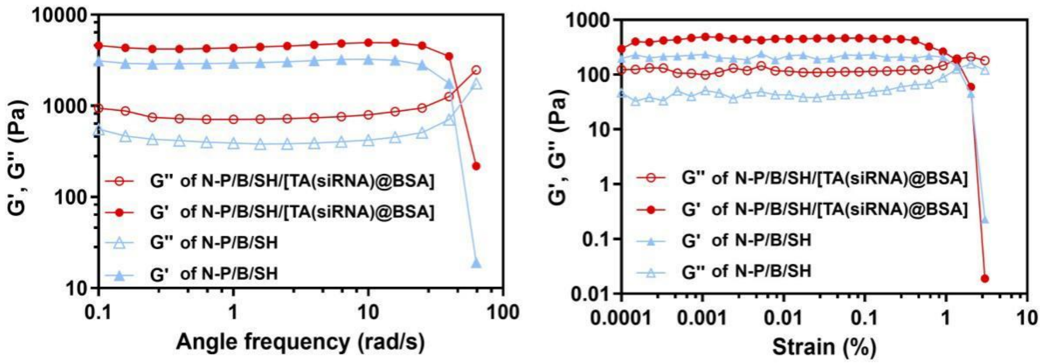

Figure S9. Rheodynamics: amplitude-strain scans of hydrogels

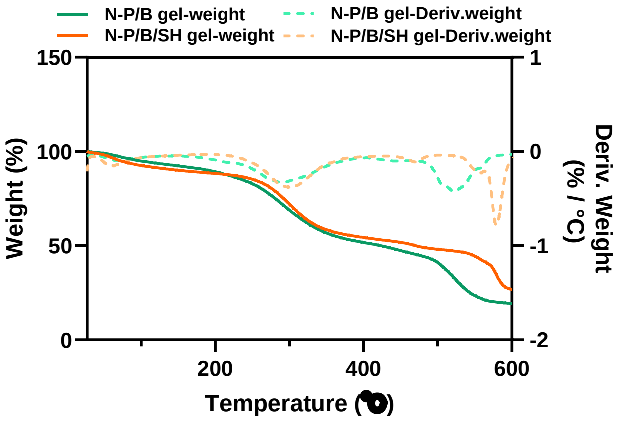

Figure S10. Thermogravimetric curves of N-P/B hydrogel and N-P/B/SH hydrogel.

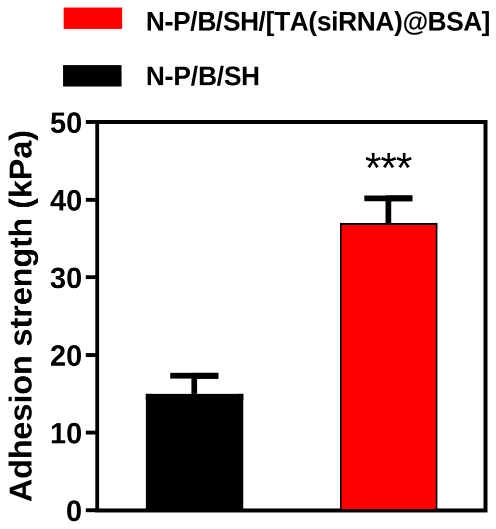

Figure S11. Adhesion strength of N-P/B/SH/[TA(siRNA)@BSA] hydrogels

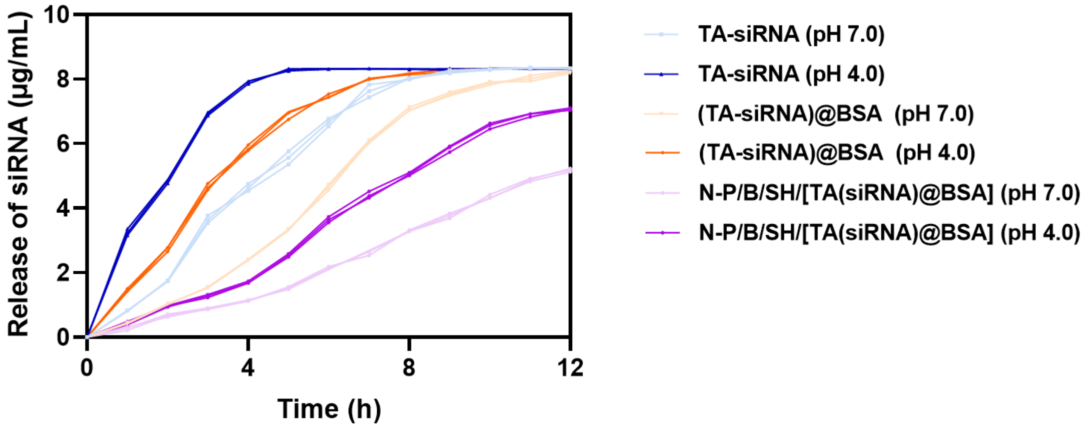

Figure S12. TA(siRNA)@BSA nanogel, TA(siRNA) nanogel, N-P/B/SH/[TA(siRNA)@BSA] hydrogel siRNA release curves at different pH.

The siRNA release rate of TA(siRNA)@BSA hydrogel was significantly lower than that of TA(siRNA), indicating that the BSA coating could enhance the structural stability of the hydrogel. In addition, the release rate of siRNA in an acidic environment (pH 4.0) was significantly higher than that in a neutral environment (pH 7.0), which suggests that it can release functional substances under acidic (wound site) conditions to exert the antioxidant, antibacterial, and scavenging efficacy of MMP-9.

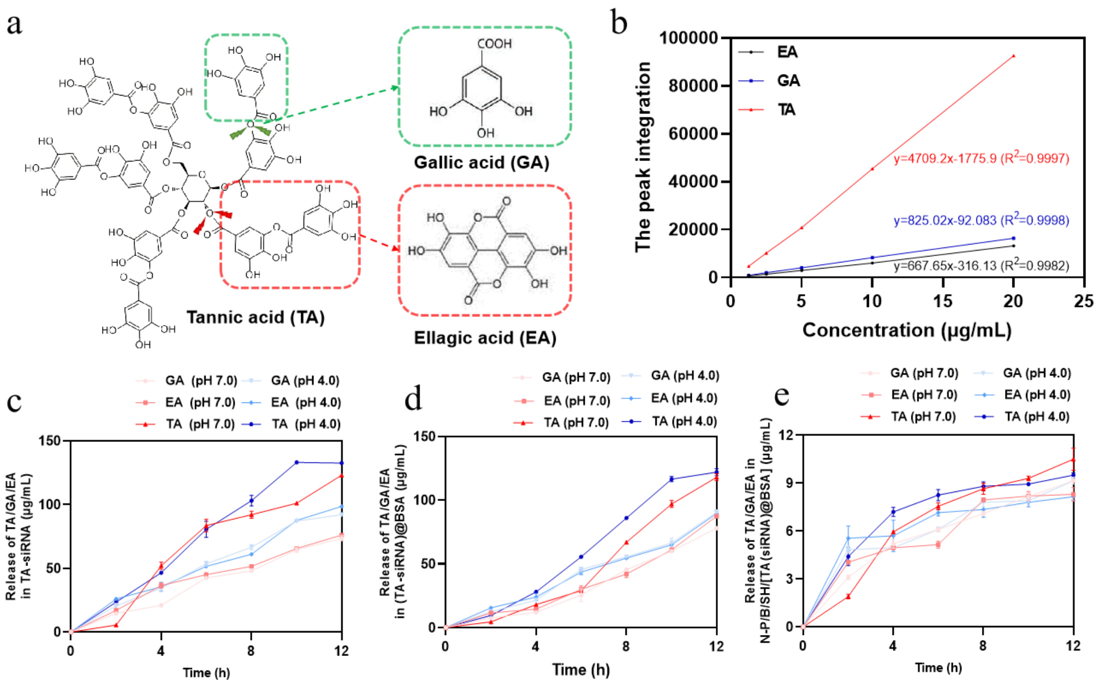

Figure S13 a) Schematic diagram of tannic acid hydrolysis; b) Standard curve of integrated peak area and TA,GA,EA concentration; TA release curves of c) TA-siRNA nanogel, d) (TA-siRNA) @BSA nanogel, and e) N-P/B/SH/[TA(siRNA)@BSA] hydrogel at different pH.

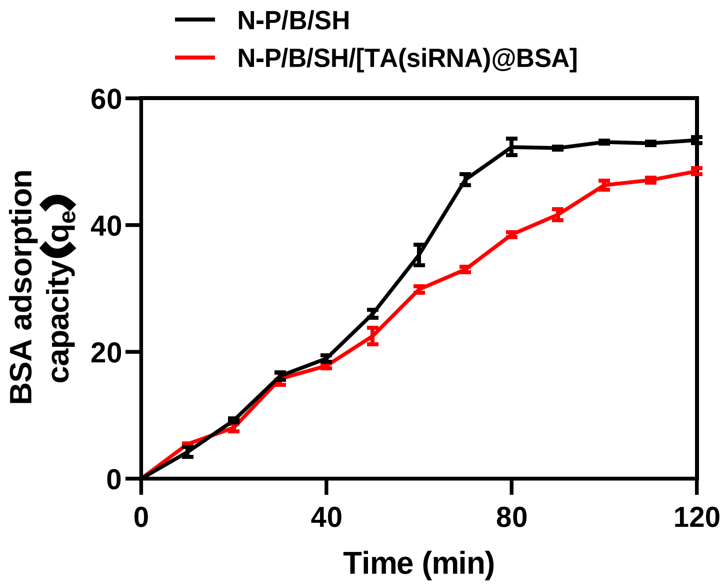

Figure S14. Kinetic curve of BSA adsorption on hydrogels

The equilibrium time required for the adsorption of BSA onto the hydrogel at pH = 7 was investigated. Figure S13 shows the kinetic curve of BSA adsorption on hydrogels. The results indicate that N-P/B/SH hydrogel adsorption reached equilibrium at approximately 80 min. In the first step, the adsorption process was very fast because

the protein molecules rapidly occupied the accessible sites of the adsorbent. As the adsorption proceeded, the rate of BSA uptake decreased and reached saturation adsorption at the end. The lower adsorption effect and adsorption rate of N-P/B/SH/[TA(siRNA)@BSA] hydrogel than that of N-P/B/SH hydrogel may be due to the fact that the TA(siRNA)@BSA nanogel occupied some of the adsorption sites.

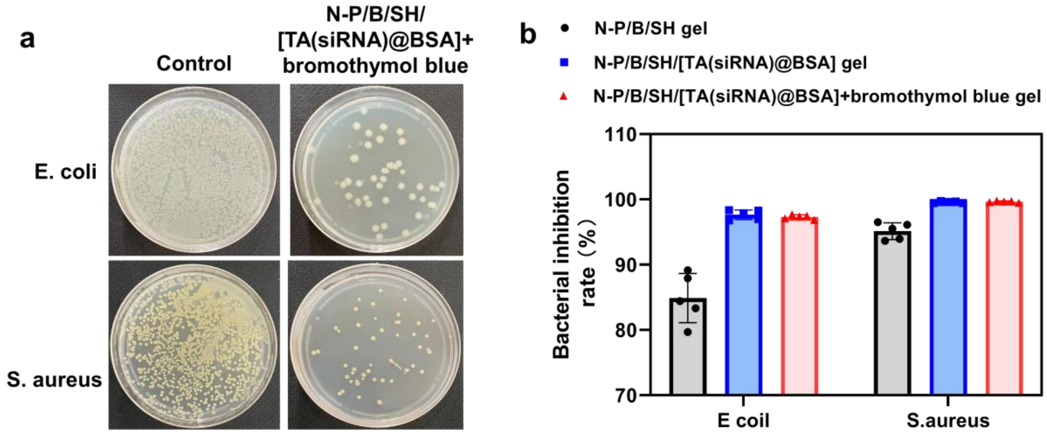

Figure S15. a) Antibacterial efficacy of hydrogel against *E. coli* and *S. aureus* by plate counting method; b) Antibacterial efficiency (statistics and calculation of data on colony forming units by plate counting method).

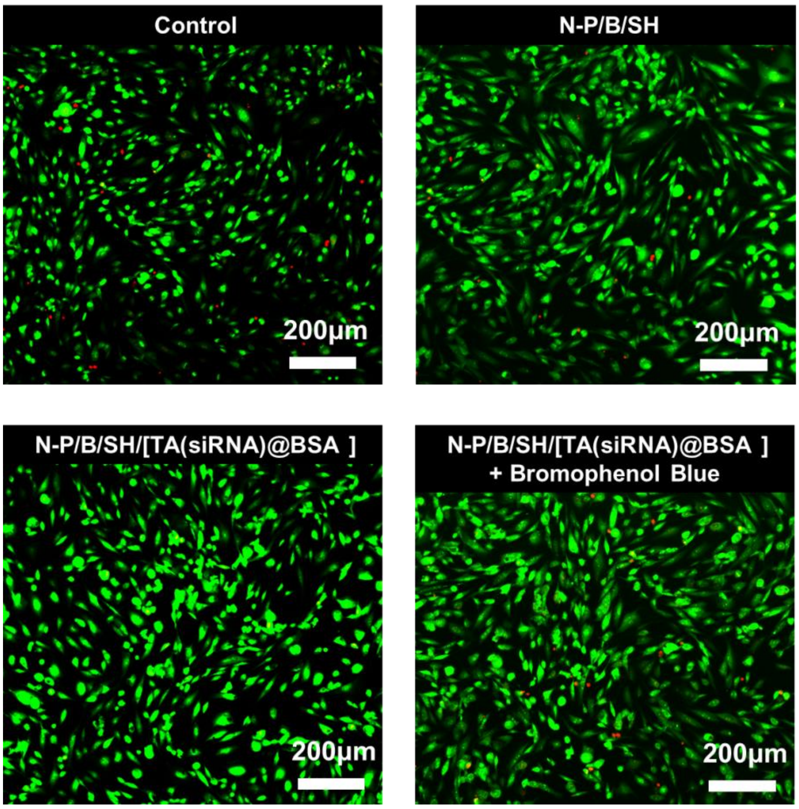

Figure S16. Live-dead cell staining (AO/EB) of HSF cells cultured with hydrogel extracts (scale bar: 200 μm)

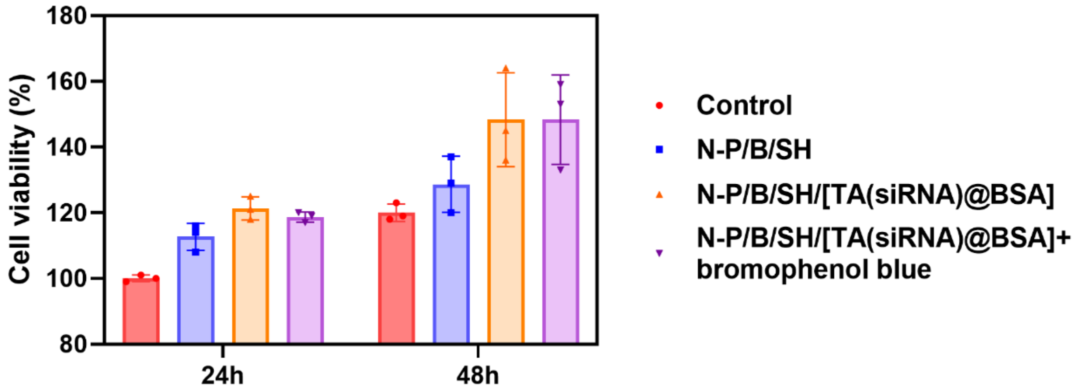

Figure S17. Cell viability of HSF cells with the hydrogel extracts;

The results obtained from experiments performed with the L929 cell model were generally similar, and all three types (N-P/B/SH; N-P/B/SH/[TA(siRNA)@BSA]; N-P/B/SH/[TA(siRNA)@BSA]+bromophenol blue) of hydrogels were not cytotoxic and were able to promote the proliferation of HSF cells and viability.

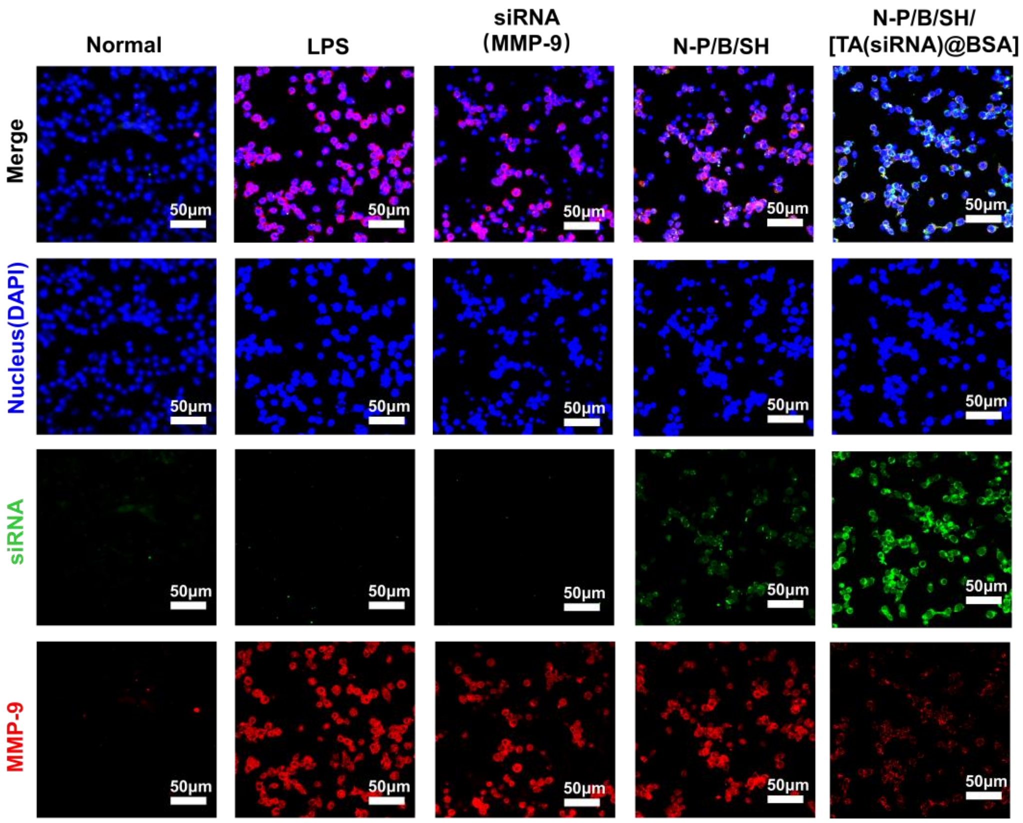

Figure S18. Efficiency of N-P/B/SH/[TA(siRNA)@BSA] on *MMP-9* gene silencing: split-channel plot of immunofluorescence staining. siRNA was labelled with 5-FAM, MMP-9 expression was assessed using immunofluorescence staining. Cell nuclei were labelled with DAPI (blue). Scale bar is 50 μm.

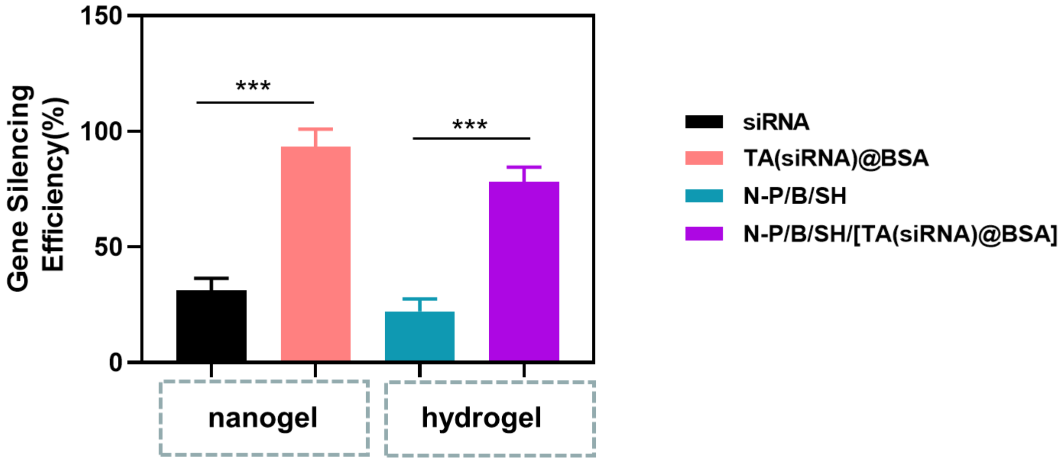

Figure S19. Gene silencing efficiency of nanogels and hydrogels(***: $p < 0.001$, $n = 5$)

The results (Figure S17-S18) showed that TA(siRNA) as well as TA(siRNA)@BSA, and N-P/B/SH/[TA(siRNA)@BSA] significantly increased the gene silencing efficiency compared to siRNA (gene silencing efficiency of about 30%), suggesting that the gene silencing efficiency of siRNA was significantly increased in the N-P/B/SH/[TA(siRNA)@BSA] hydrogel as well as TA (siRNA)@BSA nanogels

possessed functional stability. The increased gene silencing efficiency of TA(siRNA)@BSA nanogels compared to TA(siRNA) is due to the good biocompatibility of BSA as a coating as well as the transportation effect.

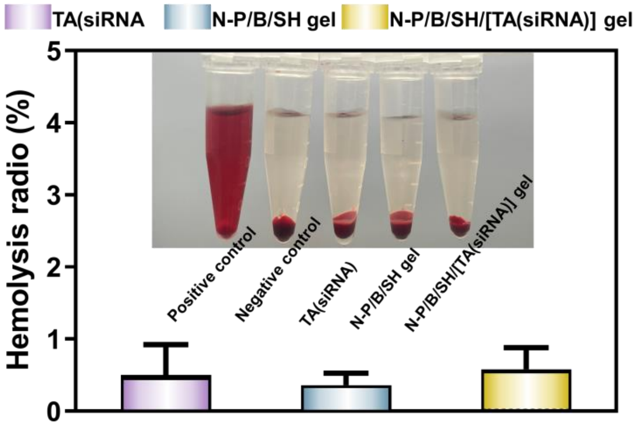

Figure S20. Hemolysis rate of TA(siRNA)@BSA nanogel, N-P/B/SH hydrogel, and N-P/B/SH/[TA(siRNA)@BSA] hydrogel

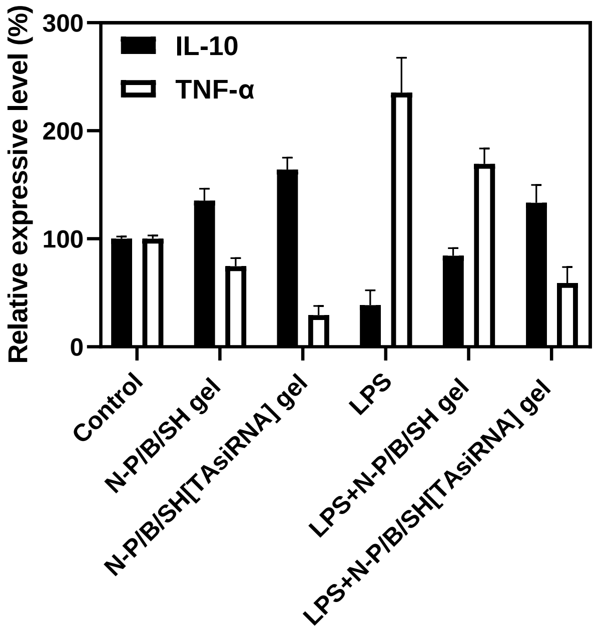

Figure S21. The levels of inflammatory cytokines (TNF-α and IL-10) secreted by cultured RAW264.7 cells were determined using enzyme-linked immunosorbent assay (ELISA) kits (Shanghai Enzyme-Linked Biotechnology Co., Ltd., Shanghai, China). We have set the control data for TNF-α and IL-10 at 100%.

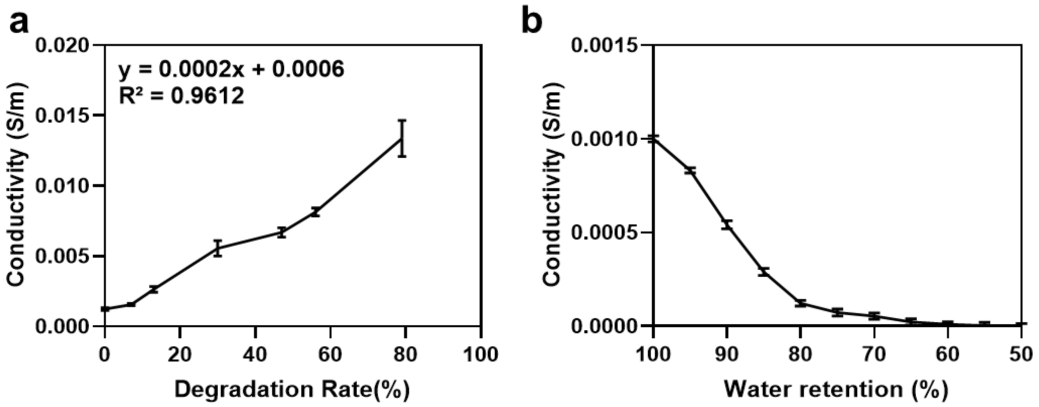

Figure S22. a) Curve of degradation and electrical conductivity b) Curve of moisture content and electrical conductivity

The electrical conductivity of hydrogels is caused by the migration of borate ions in water. The reason that hydrogel degradation leads to an increase in electrical conductivity (Fig. S21a) may be due to the collapse of the gel network or a decrease in the crosslink density, which makes the resistance to the movement of borate ions weaker, and therefore the electrical conductivity increases. In addition, the water content of the hydrogel affects the conductivity, therefore the water retention of the hydrogel at 37°Cwas tested. As shown in Fig. S21b, the decrease in water content resulted in a decrease in conductivity when the hydrogel was left at 37° C, indicating that in order to maintain the conductivity within the normal resistance of the skin, it is necessary to ensure a certain amount of water when using the hydrogel. Skin is an electrically sensitive tissue with a conductivity ranging from 1 x 10^{-5} to 0.26 S/m. Dressings with similar electrical conductivity can promote wound healing by facilitating intercellular signaling. In this study, we constructed a linear relationship between water content and electrical conductivity, which allows us to determine the range of water content that should be maintained in hydrogels in order to ensure that the electrical conductivity is in the range of skin conductivity.

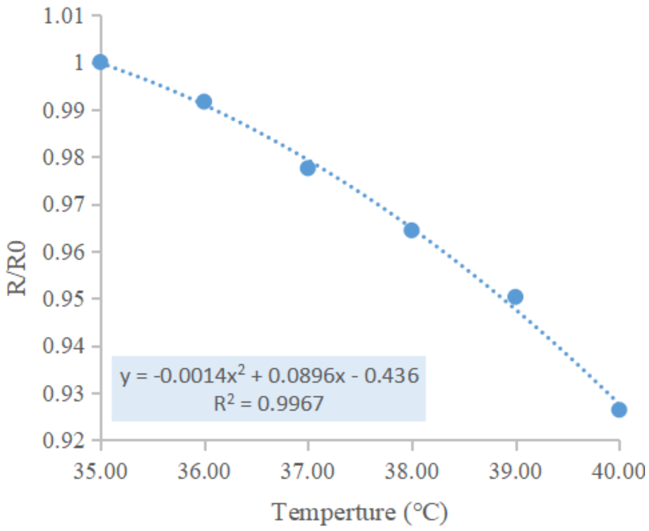

Figure S23. Standard curve for temperatures from 35℃ to 40 ℃, and a quadratic relationship between temperature and R/R0 (R^2 greater than 0.99).

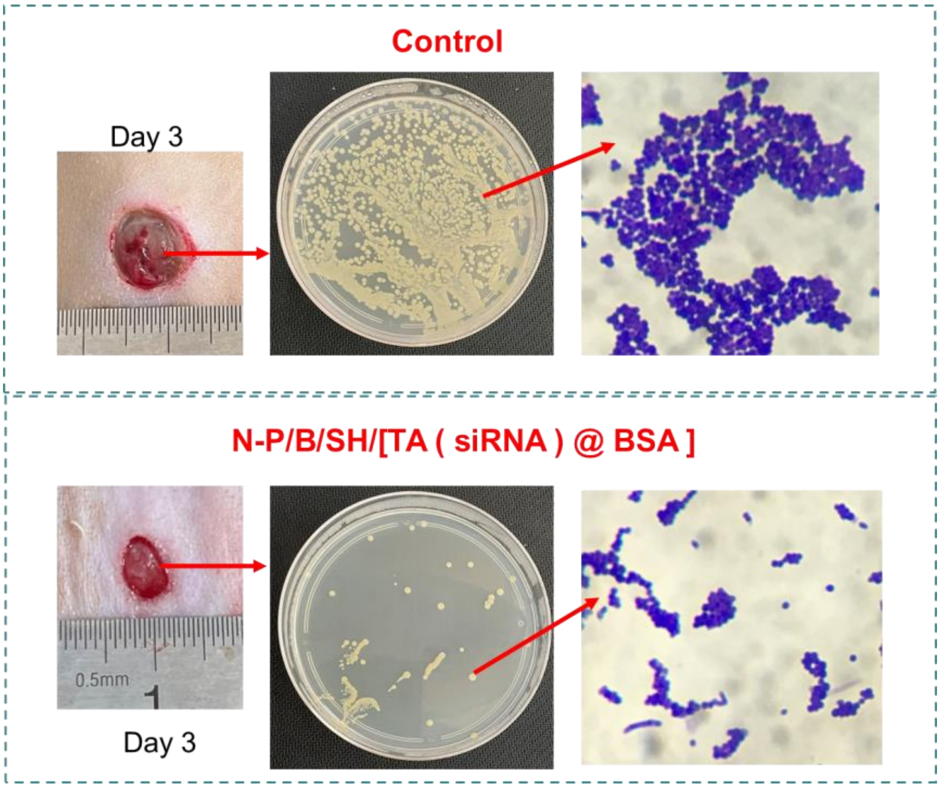

Figure S24. Antibacterial capacity of hydrogels in wounds

The wound coverings were removed after 3 days and it was observed that the wounds in the control group appeared to be infected and pus-filled, whereas the wounds in the N-P/B/SH/[TA(siRNA)@BSA] hydrogel group were clean. 100 μL of saline was added to clear the wound and the fluid was collected for plate smear. The results showed that the number of colonies in the N-P/B/SH/[TA(siRNA)@BSA] hydrogel group was

much lower than that in the control group, and the inhibition rate of the bacteria could reach more than 93% compared with the control group, and the bacteria could be recognized as Staphylococcus aureus by crystal violet staining.

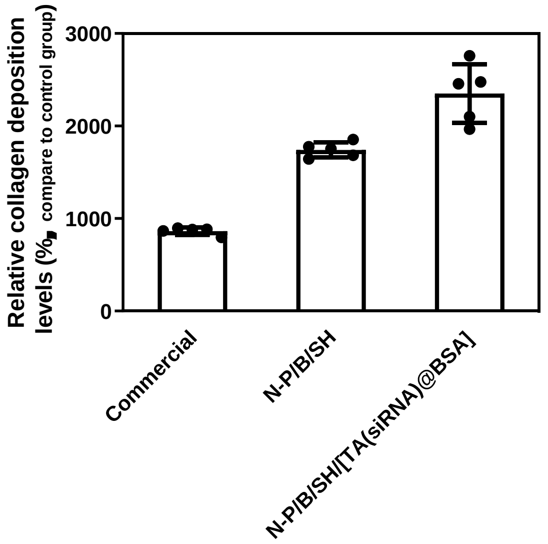

Figure S25.Quantitative data (supporting) showed that at day 10 of treatment, the relative collagen deposition levels.

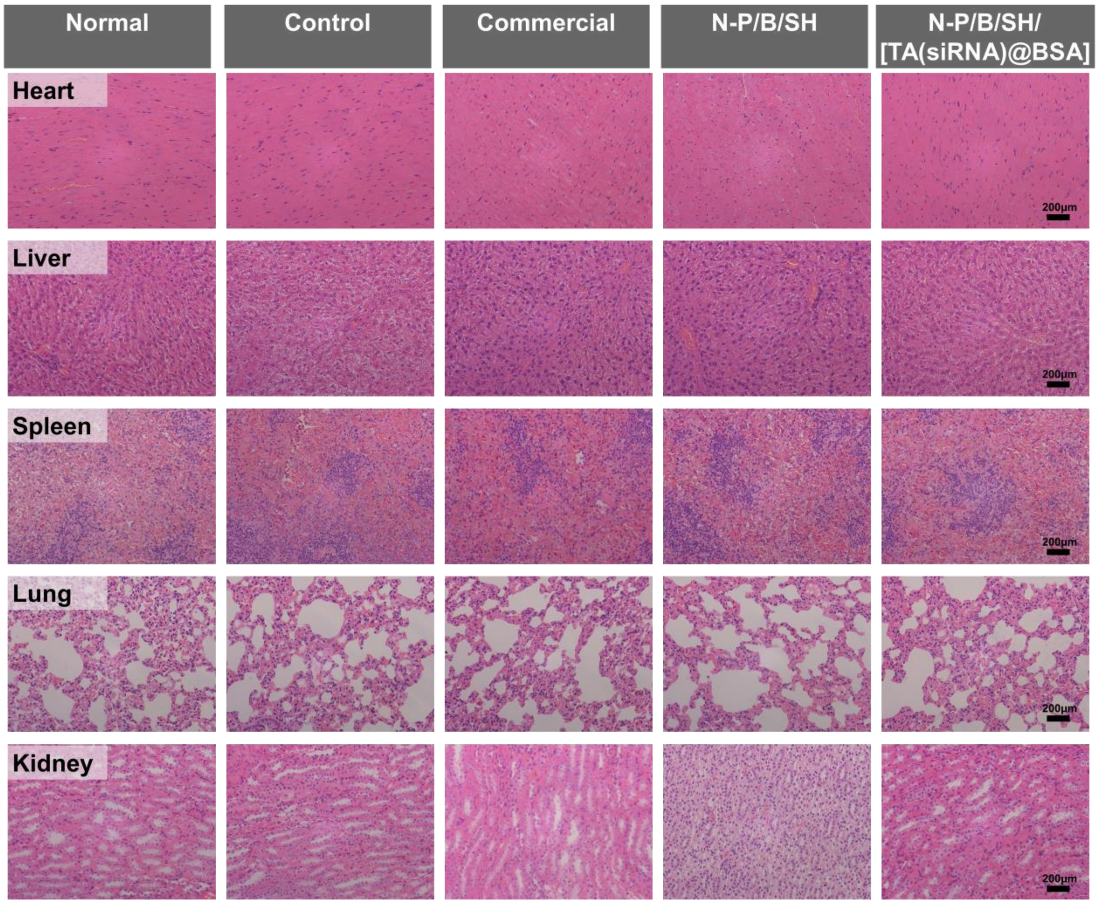

Figure S26. H&E-stained histological sections of major organs excised from rats after treatments.

Materials and methods

TEM

The prepared TA-siRNA@BSA nanogel solution was dropped on a 200 mesh copper mesh, placed at room temperature for more than 6 h to ensure water evaporation, and observed by TEM.

Particle size distribution and Zate potential

The hydrodynamic diameter and particle size distribution of the nanogels, as well as the Zate potential, were measured on a dynamic light scattering analyzer.

Gel fraction of the nanogel:

The centrifuged and filtered nanogel was lyophilized and noted as W1, and more than the centrifuged and filtered liquid was collected and lyophilized and weighed W2, and the gel fraction was calculated by Equation 1

$$Gel\ \ fraction = \frac{W_1}{W_1+W_2} \times 100\% \qquad (1)$$

Cell internalization

Macrophages were cultured in DMEM culture medium (RAW264.7), cells were collected when they were in good condition, and sterilized coverslips were placed in 6-well plates, cells were inoculated on the coverslips according to the number of 2×10^5cells/well, and 2 mL of fresh culture medium was added to wait for 6 h for cells to attach to the wall. Add siRNA and TA-siRNA@BSA nanogel (100 μL/well, siRNA group: siRNA solution with a concentration of 2 OD/mL, TA-siRNA@BSA nanogel group: TA-siRNA@BSA nanogel stock solution) to the wells respectively. Then the well plates were put into a CO_2 incubator for incubation, and the culture solution was collected separately after 12 h. The changes in fluorescence before and after incubation were determined using a fluorescence spectrophotometer, which was used to calculate the cellular uptake rate. And the coverslips were washed with PBS, then stained with DAPI solution, and then a drop of anti-fluorescence burst sealer was placed on the slides, the coverslips were placed upside down on the slides, and the pictures were taken by observing under a confocal microscope.

Gene silencing efficiency of nanogel

Macrophages (Raw264) were cultured in DMEM culture medium (RAW264.7), cells were collected when they were in good condition, and sterilized coverslips were placed in 6-well plates, cells were inoculated on the coverslips according to the number of 2×10^5cells/well, and 2 mL of fresh culture medium was added to wait for 6 h for cells to attach to the wall. Then 1 mL of LPS solution was added to induce a pro-inflammatory phenotype in macrophages (LPS concentration of 1 μg/mL). After 12 h of induction, siRNA (MMP-9) solution (100 After 12 h of induction, siRNA (MMP-9) solution (100 μL/well, 2 OD/mL), and 100 μL of TA-siRNA@BSA (MMP-9) nanogel stock solution were added to the cells in each well, respectively. 100 μL/well, 2 OD/mL), and 100 μL of TA-siRNA@BSA (MMP-9) nanogel solution were added to the cells in each well, respectively. The well plates were put into the CO_2 incubator for culture, and after 12 h, the cell crawls were stained with MMP-9 immunofluorescence staining, and the nuclei were labeled with DAPI, and then the slides were placed with a drop of anti-fluorescence burst sealer, and the coverslips were inverted on the slides, and they were observed and photographed under a confocal microscope. The experimental groupings were Control group (normal culture medium, without LPS to induce cellular inflammation), LPS model group (with the addition of LPS to induce cellular inflammation), siRNA (MMP-9) group, and TA-siRNA (MMP-9) nanogel group, with 3 parallels set up. Photographs were quantitatively analyzed by Image J software for fluorescence and gene silencing efficiency was calculated. Note that TA-siRNA@BSA nanogel should be prepared under aseptic conditions, and the siRNA used for the preparation was green fluorescence-labeled 5'FAM-siRNA. Gene silencing efficiency was determined using the following equation:

$$\text{Gene Silencing Efficiency} = \left(\frac{\text{fluorescent }\%\text{area}_{LPS} - \text{fluorescent }\%\text{area}_{samples} - \text{fluorescent }\%\text{area}_{Control}}{\text{fluorescent }\%\text{area}_{LPS} - \text{fluorescent }\%\text{area}_{Control}}\right) \times 100\%$$

(2)

Stability of nanogels under acidic conditions

The nanogel storage solution was diluted to 0.01 OD/mL, the pH was adjusted to 4.0 with 1 M HCl, and after leaving it for 6 h, 10 μL of the solution was taken and dropped on a copper grid and observed by TEM.

The cell-toxicity of nanogel

The cytotoxicity of hydrogels was determined by MTT assay. The Human Skin Fibroblasts (HSF) cells seeded at a density of 5×10^3 cell per well in 96 well plates were incubated in a humidified 5% CO_2 incubator for 24 h, followed by the addition of a mixture of nanogel stock solution and culture solution to give a nanogel concentration of 0.1 OD/mL, 0.05 OD/mL, 0.01 OD/mL. After an additional 24 h incubation, extract solutions was replaced by fresh phosphate buffered saline (PBS) containing MTT reagent. The cells were further incubated for 4 h, then the media were removed and replaced by 150 μL DMSO to dissolve the formazan crystals formed by living cells. The absorbance of each well was detected at 490 nm using a multimode microplate reader (BioTek, USA). Cell viability was determined using the following equation:

$$Cell\ viability\ (\%) = \left(\frac{A_{hydrogel}}{A_{control}}\right) \times 100\% \qquad (3)$$

where $A_{hydrogel}$ and $A_{control}$ are the absorbances of sample and control, respectively.

Modification of paramylon

2 g of paramylon was dissolved in 100 mL of dimethyl sulfoxide, and 2 g of norbornenoside and 1 g of N,N-dimethylaminopyridine were added and stirred at room temperature until dissolved, then 1 mL of triethylamine was added and the reaction was stopped after stirring for 24 h at room temperature. After dialysis purification with deionized water for 72h and vacuum freeze-drying, the paramylon (N-P) grafted with norbornene was obtained.

Cytotoxicity of different concentrations of norbornene

The cytotoxicity of hydrogels was determined by MTT assay. The HSF cells seeded at a density of 5×10^3 cell per well in 96 well plates were incubated in a humidified 5% CO_2 incubator for 12 h, followed by added with different concentrations of norbornene. After an additional 24 h, the solutions was replaced by fresh phosphate buffered saline (PBS) containing MTT reagent. The cells were further incubated for 4 h, then the media were removed and replaced by 150 μL DMSO to dissolve the formazan crystals formed by living cells. The absorbance of each well was detected at 490 nm using a multimode microplate reader (BioTek, USA). Cell viability was determined using the following equation (3).

Cryo-scanning electron microscope

The hydrogel samples were immersed in liquid nitrogen for 1 min and then removed and placed under a cryo-scanning electron microscope (PP3000T Cryoprep Transfer System (Quorum, UK), FEI Quanta 450 Environmental Scanning Electron Microscope) to examine their morphology.

Fourier transform infrared spectroscopy

Fourier transform infrared spectroscopy (Thermo Fisher Scientific, Waltham, MA, USA) spectra of hydrogel were recorded in the range of 4000-650 cm^{-1}.

Thermogravimetric analysis

To observe the thermal stability and thermal degradation of hydrogels, we used SDT-Q600 (TA Instrument) to carry out a comprehensive thermal analysis. The heating rate of 10°C/min. The temperature range was 25-800°C

Transmittance

Light transmittance of the hydrogel was acquired on a UV-VIS-NIR spectrophotometer (SHIMADAZU UV- 3600Plus) with a wavelength scanning range from 200 to 1400 nm at a scanning rate of 100 nm/min.

Swelling

Swelling test was used to determine the swelling ratio (SR) and stability of the hydrogels. The completely gelled wet hydrogels were put into 20 mL PBS (0.01 M pH 7.4) in sealed vials at 37 °C with shaking at 100 rpm. When reaching the pre-set time interval, hydrogels were taken out and the superficial water was removed using a filter paper. Following that, the hydrogels were weighed. The test was not finished until the weight of all hydrogel kept constant. SR was calculated using the following equation: SR=(100×(Wt-Wi))/Wi, where Wi and Wt represented the initial and after swelling weight, respectively.

Mechanical properties

The analysis of mechanical properties included rheological testing and compression testing. The rheological properties of the paramylon hydrogels were measured with a rheometer (Anton Paar,

MCR302) to determine the transformation between the storage modulus (G′) and loss modulus (G″) in the critical strain region and linear viscoelastic region of the hydrogel.

Tissue adhesion

The tissue-adhesion property of the hydrogels was determined by an adhesive strength test using fresh porcine skin as the tissue model. Briefly, the skin tissue was cut into a 20 mm × 80 mm rectangle and then immersed into phosphate-buffered saline (PBS) before use. Five hundred microliters of hydrogel solutions were applied onto the surface of porcine skin and another skin was put onto the hydrogel solution. The adhesive area was 20 mm × 20 mm. Subsequently, the porcine skin was warmed at 37°C for 1 h before the test. The sample was lap-shear tested to failure at a speed of 10 mm/min on the tensile testing machine (INSTRON 5565, Instron, Norwood, MA, USA). The adhesive capacity was calculated from the maximum modulus over the area of the adhesive overlap.

Release of siRNA

Steps of siRNA release experiments in nanogel: nanogel storage solution was diluted to 0.25OD/mL, and the pH was adjusted to 4.0 and 7.0, respectively. siRNA concentration was measured at each time point using the nanodrop instrument. Steps of siRNA release experiments in hydrogel: PBS buffer with pH 4.0 and 7.0 was configured, and hydrogel (0.1g/mL) was added. Then the siRNA concentration in the solution was measured at each time point using the nanodrop instrument.

Release of TA

Reversed-phase liquid chromatography (RP-LC) (Agilent 1200, Santa Clara, CA, USA) was used to analyze the degradation of TA. Tris buffer (10×10^{-3} M, pH 7.4) was used as eluent. The gradient of the mobile phase was as follows: 5% acetonitrile containing 0.1% trifluoroacetic acid (TFA) with a gradient of 64% for the first 20 min, which was further increased to 95% for the next 10 min and then decreased to 5% for the next 15 min (i.e., 20-30 min). The retention time was determined using a UV detector (270 nm). The flow rate for both chromatograms was 0.5 mL min^{-1}. Standard curves of integrated peak area and TA, GA, EA concentrations were first determined and then the amount of TA released from the hydrogel was calculated from the standard curves. The experimental groups were: TA-siRNA nanogel, TA(siRNA)@BSA nanogel, N-P/B/SH/[TA(siRNA)@BSA] hydrogel.

Experimental environment: TA release in experimental groups at different pH (pH 4.0 and pH 7.0). The assay indicator is the amount of TA, GA, EA in different time period. Steps of TA release experiments in nanogel: nanogel storage solution was diluted to 0.25OD/mL, and the pH was adjusted to 4.0 and 7.0, respectively. Steps of TA release experiments in hydrogel: PBS buffer with pH 4.0 and 7.0 was configured, and hydrogel (0.1g/mL) was added.

BSA adsorption on hydrogel

Adsorption of proteins from aqueous solutions using hydrogels (BSA) Approximately 0.1 g of dried hydrogel was placed in 20 mL of BR buffer solution (different pH) containing 4.0 mg/mLof BSA and equilibrated for solubilization at 30 ℃. A portion of the solution was removed and the concentration was measured by UV spectrophotometry at 279 nm. A portion of the BSA solution was removed and the concentration was measured at 279 nm using a UV spectrophotometer. The amount of BSA adsorbed on the hydrogel (qe) was calculated by the following equation. where qe is the amount of BSA adsorbed (mg/g), Ci is the initial concentration, Ce is the equilibrium concentration of the solution, Vt is the volume of the solution, and m is the mass of the dry hydrogel.

$$q_e = \frac{(c_i - c_e)}{m} V_t \tag{4}$$

In vitro cytotoxicity

The cytotoxicity of hydrogels was determined by MTT assay. To obtain extract solutions, hydrogels were immersed in culture medium with the concentration of 100 mg/mL for 3 d. The L929 cells seeded at a density of 1×10^4 cell per well in 96 well plates were incubated in a humidified 5% CO_2 incubator for 24 h, followed by treated with extract solutions. After an additional 24 h and 48h incubation, extract solutions was replaced by fresh phosphate buffered saline (PBS) containing MTT reagent. The cells were further incubated for 4 h, then the media were removed and replaced by 150 μL DMSO to dissolve the formazan crystals formed by living cells. The absorbance of each well was detected at 490 nm using a multimode microplate reader (BioTek, USA). Cell viability was determined using the following equation (3).

The cell viability of HSF cells was tested in the same way, with a cell density in 96-well plates of 5 $\times 10^3$.

Live/dead (L/D) cell staining analysis

The viability of L929 cell on the tested materials was evaluated using a Live/Dead cell staining and fluorescence microscopy. The hydrogels were sterilized by Co60 irradiation (72 h, 10 kGy) and then hydrogels were immersed in culture medium with the concentration of 100 mg/mL for 3 d. The 2×10^5 of cells were seeded in a six-well plate, and extract culture medium was added. Cells were then placed in an incubator at 37°C with 12h, the cells were washed with PBS to remove unattached cells. Live/Dead cell viability kit for mammalian cells was used for Live/Dead cell staining to evaluate the cell growth. We used microscopy to observe the stained L/D cells. The tested hydrogels included N-P/B/SH hydrogel and N-P/B/SH/[TA(siRNA)@BSA] hydrogel.

The cell viability of HSF cells was tested in the same way, with a cell density in 6-well plates of 1×10^5.

Gene silencing efficiency of hydrogels

Macrophages (Raw264) were cultured in DMEM culture medium (RAW264.7), cells were collected when they were in good condition, and sterilized coverslips were placed in 6-well plates, cells were inoculated on the coverslips according to the number of 2×10^5cells/well, and 2 mL of fresh culture medium was added to wait for 6 h for cells to attach to the wall. Then 1 mL of LPS solution was added to induce a pro-inflammatory phenotype in macrophages (LPS concentration of 1 μg/mL). After 12 h of induction, the hydrogel extract (100 mg/mL, serum content 1v/v%) were added to the cells in each well, respectively. The well plates were put into the CO_2 incubator for culture, and after 12 h, the cell crawls were stained with MMP-9 immunofluorescence staining, and the nuclei were labeled with DAPI, and then the slides were placed with a drop of anti-fluorescence burst sealer, and the coverslips were inverted on the slides, and they were observed and photographed under a confocal microscope. The experimental groupings were Control group (normal culture medium, without LPS to induce cellular inflammation), LPS model group (with the addition of LPS to induce cellular inflammation). The tested hydrogels included N-P/B/SH hydrogel and N-P/B/SH/[TA(siRNA)@BSA] hydrogel, with 3 parallels set up. Photographs were quantitatively analyzed by Image J software for fluorescence and gene silencing efficiency was calculated. Note that TA-siRNA@BSA nanogel should be prepared under aseptic conditions, and

the siRNA used for the preparation was green fluorescence-labeled 5'FAM-siRNA. Gene silencing efficiency was determined using the following equation (2).

Cell migration assay

Cell migration experiments simulated wound healing *in vitro*. The L929 cells were cultured in hydrogel extract (100 mg/mL, serum content 1v/v%) and the control group was cultured in a normal medium (serum content 1%). The experiments were divided into two groups: N-P/B/SH hydrogel group and N-P/B/SH/[TA(siRNA)@BSA] hydrogel group. L929 cells (1×10^6 cells/well) were seeded in a six-well plate. A scratched cell monolayer (with a sterile 200-µL pipette tip) mimicked an incision wound on the conductive glass, and the monolayer was washed with PBS to remove cellular debris. The healing process was monitored by photographing a scratch at 0 h and 24 h using a microscope. The scratched area was analyzed with ImageJ (x64). The cell migration rate was obtained as follows:

$$cell\ migration\ rate\ (\%) = \frac{A_0 - A_t}{A_0} \times 100\% \qquad (4)$$

where A_0 and A_t are the initial scratch area and the scratch area after a period of culture, respectively.

Tube formation experiment

The matrix gel was placed in a 4°C refrigerator overnight and thawed 24 h in advance for spare parts, and the lance tip and well plate required for the experiment were placed in a 4°C refrigerator for cooling. Place the 24-well plate on an ice box, add 300 µL of matrix gel to each well of the 24-well plate, and place the plate in an incubator at 37°C for 30 minutes. Cultured HUVEC cells were digested and collected, and 200 µL of HUVEC cell suspension was added to a 24-well plate (2×10^4 cells per well) to inoculate onto the cured matrix gel substrate. N-P/B/SH hydrogel and N-P/B/SH/[TA(siRNA)@BSA] hydrogel extracts (100 mg/mL) were added to the experimental group, and blank medium was added to the blank group. After 6 hours of incubation (5% CO2, 37°C), cells were imaged using a microscope and the number of branches per square millimeter was measured using ImageJ software.

Antibacterial test

The morphology of bacteria after different treatments was observed by scanning electron

microscopy (SEM). Bacterial suspensions from different treatments were placed on coverslips and fixed, then dehydrated with gradients of 50%, 70%, 90%, 95% and 100% ethanol, respectively. Finally, the samples were sputter plated with gold for SEM observation.

The membrane structure of the bacteria was detected by TEM. Bacteria from different hydrogel treatments were collected, and centrifuged at 6000 rpm for 5 min, and the supernatant was discarded. Subsequently, the collected bacteria were resuspended in transmission electron microscopy fixative, and after fixation, the samples were incubated in osmium tetroxide (1%) at 4°C overnight and then washed three times with PBS. After dehydration through a series of ethanol gradients (50, 70, 80, 90, 95, and 100%), and further embedded in paraffin for ultrathin sectioning. After drying and staining with uranyl acetate, the sections were placed on a bare copper grid and observed with a transmission electron microscope.

Antioxidant experiment

To determine the ability of the hydrogels to scavenge $\bullet O_2^-$. Hydrogel was mixed with 12 mL of Tris-HCl (50 mM, pH 8.1), and 2 mL of pyrogallol (3 mM) was subsequently added to the mixture, which was then allowed to stand for 5 min in the dark. The groups were N-P/B/SH hydrogel and N-P/B/SH/[TA(siRNA)@BSA] hydrogel samples (0.1 g), and ascorbic acid (100 μmol, 100μL). The reaction was stopped by adding 4 mL of hydrochloric acid (1 M) to each group, and the absorbance at 299 nm was measured. The $\bullet O_2^-$ scavenging effect was calculated:

$$Scavenging\ effect\ (\%) = \left(\frac{A_{control} - A_{hydrogel}}{A_{control}}\right) \times 100\% \qquad (5)$$

Scavenging ability of hydrogels for •OH: $FeSO_4$ (1.0 mL, 9.0 mM), hydrogel (0.1 g) and salicylic acid (1.0 mL, 9.0 mM) were placed into a centrifuge tube, H_2O_2 was added to the centrifuge tube at 37 ℃ for 30 min, and the absorbance at 510 nm was measured. The groups were N-P/B/SH hydrogel and N-P/B/SH/[TA(siRNA)@BSA] hydrogel samples (0.1 g), and ascorbic acid (100 μmol, 100μL). The •OH scavenging effect was calculated using the following equation:

$$Scavenging\ effect\ (\%) = \left(\frac{A_{hydrogel}}{A_{control}}\right) \times 100\% \qquad (6)$$

DPPH scavenging assay: DPPH (3.0 mL, 100 μM) and dispersion of N-P/B/SH hydrogel and N-P/B/SH/[TA(siRNA)@BSA] hydrogel samples (0.1g) in methanol were stirred and incubated in a

dark place for 15 min. The mixture was stirred and allowed to stand in the dark for 15 min. absorbance values at 517 nm were measured by UV-Vis spectrophotometer. the clearance of DPPH was calculated according to equation 3, where A_{blank}, $A_{hydrogel}$ are the absorbance values of blank (DPPH/ethanol solution) and hydrogel (DPPH/ethanol + hydrogel) at 517 nm, respectively.

ABTS radical cation ($ABTS^{\cdot+}$) scavenging assay: $ABTS^{\cdot+}$ was produced by the reaction of 7.4 mM ABTS solution with 2.6 mM potassium persulfate ($K_2S_2O_8$), and the mixture needed to be placed in the dark at room temperature for 24 h. Before use, the $ABTS^{\cdot+}$ solution was adjusted with ultrapure water to an absorbance at 734 nm of 0.70 ± 0.02. Then, the N-P/B/SH hydrogel and N-P/B/SH/[TA(siRNA)@BSA] hydrogel samples (0.1g) were mixed with 3 mL of $ABTS^{\cdot+}$ solution and incubated in the dark for 20 min. the absorbance of the solution at 734 nm was measured. the $ABTS^{\cdot+}$ scavenging effect was calculated according to equation 4, where A_{blank}, $A_{hydrogel}$ are the absorbance of $ABTS^{\cdot+}$ solution at 734 nm before and after mixing with the sample, respectively.

$$\text{DPPH scavenging } (\%) = \left(\frac{A_{blank} - A_{hydrogel}}{A_{blank}}\right) \times 100 \quad (7)$$

$$\text{ABTS}^{\cdot+} \text{ scavenging } (\%) = \left(\frac{A_{blank} - A\ _{hydrogel}}{A_{blank}}\right) \times 100 \quad (8)$$

Conductivity

The hydrogel was uniformly filled in a mold 50 mm (length) x 10 mm (width) x 0.25 mm (thickness) rectangular mold, copper sheets were placed on both sides of the mold, and two wires were connected to a multi-channel touch screen digital meter to detect the resistance value. The conductivity was calculated as shown in Equation (9), where L is the length and S is the contact area between the hydrogel and the copper sheet.

$$\sigma = \frac{L}{RS} \quad (9)$$

Relationship between degradation rate and electrical conductivity

The prepared hydrogel was placed in a degradation solution (PBS buffer at pH 7.0 with lysozyme), and a piece of hydrogel was taken out every hour and tested for electrical conductivity, and the hydrogel with tested electrical conductivity was freeze-dried and the degradation rate of the hydrogel was calculated. The formula for calculating the degradation rate is shown below, where

$W_{initial}$ is the mass of the hydrogel after freeze-drying before degradation and $W_{degrade}$ is the mass of the hydrogel after degradation after freeze-drying.

$$\text{De} = \left(\frac{W_{initial} - W_{degrade}}{W_{initial}}\right) \times 100\% \qquad (10)$$

Relationship between moisture content and electrical conductivity

The water retention properties of hydrogels were tested by measuring the mass of hydrogels at different temperatures versus time. The hydrogel samples were placed at a constant temperature of 37° C. A piece of hydrogel was removed every 2 h and tested for electrical conductivity, in addition to weighing the wet weight of the hydrogel. The water retention was calculated by equation 11. $W_{Initial\ water}$ is the weight of water in the initial hydrogel, $W_{Initial\ gel}$ is the weight of the initial hydrogel, and W_{gel} is the weight of the hydrogel at different times.

$$\text{Water Retention}(\%) = \frac{W_{Initialwater} - (W_{Initialgel} - W_{gel})}{W_{Initialwater}} \times 100\% \qquad (11)$$

In vivo antibacterial assay

A whole skin injury model was established, and 10 μL of bacterial solution (S. aureus concentration: 1×10^7 CFU/mL) was added to the wound, and after five minutes, the wound was covered with N-P/B/SH/[TA(siRNA)@BSA] hydrogel dressing with gauze to prevent dislodgement, while the control group was covered with gauze only. the wound covering was removed after 3 days, and the wound was clear by adding 100 μL of saline and the fluid was collected for plate smear and microscopic examination of crystalline violet staining. After 3 days, the wound coverings were removed, 100 μL of saline was added to clear the wound and the fluid was collected for plate smears and microscopic examination of bacterial colonies with crystal violet staining.
